# Acknowledgment to Reviewers of *Nanomaterials* in 2021

**DOI:** 10.3390/nano12040667

**Published:** 2022-02-17

**Authors:** Nanomaterials Editorial Office

**Affiliations:** MDPI AG, St. Alban-Anlage 66, 4052 Basel, Switzerland

Rigorous peer-reviews are the basis of high-quality academic publishing. Thanks to the great efforts of our reviewers, *Nanomaterials* was able to maintain its standards for the high quality of its published papers. Thanks to the contribution of our reviewers, in 2021, the median time to first decision was 15 days and the median time to publication was 33 days. The editors would like to extend their gratitude and recognition to the following reviewers for their precious time and dedication, regardless of whether the papers they reviewed were finally published:
Abakumov, Maxim A.Liu, DanAbate, LorenzoLiu, Der-zenAbbes, BoussadLiu, HaihuAbbina, SrinivasLiu, HaoAbbott, StevenLiu, Hsien-KuangAbbrent, SabinaLiu, HuAbd El-Mageed, TaiaLiu, HuiAbdel-Aziz, H.M.M.Liu, JunminAbdelhamid, Hani NasserLiu, Keng-KuAbdel-Mageed, AliLiu, KuanAbdollahifar, MozaffarLiu, LiweiAbiev, RufatLiu, MeiAbitbol, TiffanyLiu, PeijiangAblat, AbdulezizLiu, PorunAbou-Hassan, AliLiu, QiangAbramova, AnnaLiu, RuijiangAbramović, BiljanaLiu, ShudeAbramovich, HaimLiu, Shyh-JiunAbrantes, Ana MargaridaLiu, Ting-YuAbu-Hamdeh, Nidal H.Liu, XianhuAbujetas, Diego R.Liu, XianjieAdachi, SadaoLiu, XinyingAdamiano, AlessioLiu, XuanyongAdamski, AndrzejLiu, YanghuiAdeagbo, Waheed A.Liu, YingAdeel, MuhammadLiu, YuAdegoke, OluwasesanLiu, YunAdhikari, RajanLiu, ZhitianAdhikari, RajdeepLivache, ClémentAdil, Syed FarooqLlorens, José ManuelAdlhart, ChristianLloveras, PolAfanasev, AndreiLo Faro, Maria JosèAfrasiabi, ZahraLo Sciuto, GraziaAfsari, MortezaLo, Yu ChiehAgarwala, ShwetaLocatelli, MarcelloAgnoli, StefanoLocci, Antonio MarioAguila, BrianaLockett, MatthewAgustin, DominiqueLofland, SamAhmad, KawsarLoncaric, IvorAhmadivand, ArashLonghi, MariangelaAhmed, MaryaLongo, AngelaAhmed, SamehLongo, SandroAhmmad, BashirLonjon, AntoineAhn, DoyeolLopes, AndréAhn, SeokhoonLopez Anton, RicardoAhn, Yeong HwanLópez Romero, J. M.Ai, KelongLópez, David MarreroAidun, DaryushLopez, FrancescoAigouy, LionelLopez, GartzenAikawa, ShinyaLópez, Miguel ÁngelAirinei, AntonLopez, ReneAissa, BrahimLopez-Anton, M. AntoniaAivaras, KareivaLopez-Garzon, RafaelAizikovich, SergeiLópez-Ortega, AlbertoÅkermark, BjörnLópez-Periago, Ana M.Akhatov, IskanderLopez-Ramon, Maria VictoriaAkhavan, OmidLorente, SylvieAkhtar, SultanLorusso, FeliceAkimov, AndreyLos, MarcinAkitsu, TakashiroLoschenov, Victor B.Akiyama, ToruLosi, PaolaAlabri, MohammedLosic, DusanAlamri, SabriLosurdo, MariaAlavi, SamanLota, KatarzynaAlbarracin, RicardoLotnyk, AndriyAlbella, PabloLott, DieterAlbero, JosepLour, Wen-ShiungAlberto, VertovaLoureiro, Francisco J.A.Albini, AngeloLovell, JonathanAlbizuri, JosebaLow, NicholasAlbonetti, CristianoLoza, KaterynaAlcaide Monterrubio, FranciscoLozac’h, MickaëlAlen, BenitoLu, ChenyangAl-Enizi, Abdullah M.Lu, JianguoAleshin, AndreyLu, Kuo-ChangAlessandro, MattoniLu, LehuiAlexander-Webber, JackLu, LeiAlexiou, ChristophLu, MinghuaAlfano, MarcoLu, MinghuiAlfè, MichelaLu, TianliangAlfieri, Maria LauraLu, Wan-LiangAlgarra González, ManuelLu, XingxuAli, GhulamLu, YanAli, GomaaLuan, Pi-GangAli, ImranLuan, YuxiaAli, MohdLucaciu, Constantin MihaiAli, Moustafa R. K.Lucas, Ivan T.Ali-Boucetta, HaneneLuciani, GiuseppinaAlikin, DenisLučić, BonoAl-Kheetan, MazenLuckham, PaulAllen, AndrewLüdge, KathyAllen, Norman S.Luin, StefanoAllizond, ValeriaLukaszewicz, JerzyAlloin, FannieLukoyanov, AlexeyAlluri, Nagamalleswara RaoLukšič, MihaAlmásy, LászlóLukyanov, Pavel A.Almeida, Bernardo GonçalvesLuliński, PiotrAlmquist, CatherineLuliński, SergiuszAlonso Masa, JavierLuna, CarlosAlonso, José AntonioLunelli, LorenzoAlonso-Vante, NicolasLungu, CristianAlpuche-Aviles, Mario A.Lunov, OlegAltucci, CarloLuo, DanAlvarez, GonzaloLuo, JianbinÁlvarez-Prado, Luis M.Luo, JianjunÁlvarez-Rodríguez, JesúsLuo, LiangAlves, PatríciaLuo, LiqiangAly, Arafa HLuo, LiuAmabili, MarcoLuo, XiangangAmade, RogerLuo, YanghuiAmador, UlisesLuo, YuAmanianpong, Prince NanaLuo, YuanfangAmarie, DragosLuo, ZhipingAmemiya, KentaLuongo, GiuseppeAmendola, VincenzoLupa, LaviniaAmer, MohLupi, Federico FerrareseAmoruso, SalvatoreLuttge, ReginaAmovilli, ClaudioLützenkirchen-Hecht, DirkAn, SangminLuxbacher, ThomasAnahory, YonathanLv, HualiangAnanyev, MaximLv, KangleAnastasescu, MihaiLvov, YuriAndo, YoshitoLvova, LarisaAndón, Fernando TorresLwin, Thinzar M.Andrade, LuísaLyer, StefanAndrade, Marta AmaralLykakis, Ioannis N.Andrade, Suzana M.Lynch, IseultAndreev, Dmitrii E.Lyon, R. E.Andrei, SarychevLyons, Daniel MarkAndreoli, CristinaLysova, Anna A.Andreou, ChrysafisLyu, MiaoqiangAndronic, LuminitaMa, Chih-MingAndrukhov, OlehMa, GangAndrulevicius, MindaugasMa, HonganAng, Yee SinMa, XiaoliAngelici, Robert J.Ma, XingmaoAngelo, AngeliniMa, YinjiAngelov, BorislavMa, ZengshengAngsantikul, PavimolMa, ZhenAnikeev, S. G.Ma, ZhuoranAnitas, Eugen MirceaMaas, MichaelAnni, MarcoMacak, JanAnnunziata, MarcoMaćkowski, SebastianAnnunziata, OnofrioMacutkevič, JanAnsón Casaos, AlejandroMączka, MirosławAntipov, Oleg L.Madden, DavidAntoine, RodolpheMadhyastha, HarishkumarAntolini, ErmeteMadison, Alexey E.Antonelli, AntonellaMadruga, SantiagoAntonio, CarellaMaduraiveeran, G.Antonioli, DiegoMaeda, KoujiAntonova, Irina V.Maeda, NobuoAnttu, NicklasMaeng, Han-JooAntunes, AlexandraMaenosono, ShinyaAnwar, ZahidMaffei, NicolaAoki, HiroyukiMagalhães, RicardoAparicio Rebollo, Francisco JavierMaggioni, DanielaAparicio, Francisco J.Magkoev, Tamerlan T.Apetrei, ConstantinMagna, GabrieleApostoluk, Aleksandra (Alexandra)Magnaudeix, AmandineAprili, MarcoMagrez, ArnaudAprodu, IulianaMagro, MassimilianoAquilano, DinoMaharjan, PukarAquino, Adelia J. A.Mahata, ChandreswarArai, RyoichiMahmood, NasirAraújo, Alberto N.Mahmoudi, MortezaArauzo, A.Mahon, Peter J.Arciello, AngelaMahy, Julien G.Arefi, MohammadMaimistov, Andrei I.Arena, AntonellaMaiuri, PaoloArena, MaurizioMajerz, IrenaArgurio, PietroMakoś, PatrycjaAriga, KatsuhikoMakris, Antonios M.Arinstein, ArkadiMaksimov, Dmitrii N.Arjmandi, RezaMakvandi, PooyanArkadiusz, CiesielskiMalaika, AnnaArkas, MichaelMalandrino, GraziellaArmandi, MarcoMalavasi, LorenzoArnould, MarkMale, David K.Arnusch, Christopher J.Maleki, SolmazArrabito, Giuseppe DomenicoMali, SawantaArrieta, Marina P.Malik, RituArruebo, ManuelMalik, SharaliArsenin, Aleksey V.Malikan, MohammadArshad, MuhammadMalinauskas, MangirdasArslan, HakanMalinowska, ElżbietaArtem’ev, Alexander V.Malinowski, RafałArthanareeswaran, GMalka, DrorArul, Narayanasamy SabariMalucelli, GiulioAsadi, AminMaluta, SergioAsakawa, NaokiMammeri, FaynaAsandei, AlinaManaila-Maximean, DoinaAshuri, MaziarManakhov, AntonAsín, LauraManakov, AndreyAskes, SvenManas, MiroslavAsokan, ArunchanderManciu, FeliciaAssmann, MarcManconi, MariaAstrova, EkaterinaMandal, SarthakAta, SeisukeManderville, Richard A.Atabaev, Timur Sh.Mandić, VilkoAtanase, LeonardMandzhieva, Saglara S.Atchudan, RajiManeiro, MarcelinoAtia, HananManera, Maria GraziaAtienzar, PedroMani Rajan, Murugan SenthilAtkinson, IrinaManière, CharlesAtobe, MasakazuManikandan, ElayaperumalAtochin, DmitriyManini, PaolaAtrei, AndreaManna, SantanuAttri, PankajMannell, HannaAtuchin, VictorManno, Daniela ErminiaAudebert, PierreMantione, DanieleAuger, Maria AManzhos, SergeiAuslender, MarkMao, ChunAvgeropoulos, ApostolosMao, YanchaoAviani, IvicaMaqbool, MuhammadAvramescu, Sorin MariusMaqsood, Muhammad Aamer Avramopoulos, AggelosMarabelli, FrancoAwni, RashaMarcelo, GemaAwual, Md. RabiulMarchenko, DmitryAxet, M. RosaMarchesan, SilviaAymonier, CyrilMarconato, NicoloAytac, ZeynepMarconcini, PaoloAyub, Prof. KhurshidMarcos, RicardAyude, Maria AlejandraMarcu, Ioan CezarAzevedo, SérgioMarcus, Matthew A.Aziz, MustafaMarek, TkaczB. Carda Castelló, JuanMarenzi, GaetanoBabilas, RafałMargaritis, LukasBabincová, MelániaMaria, Vera LúciaBabu, BathulaMariani, PaoloBabula, PetrMariappan, Vimal KumarBaccarani, GiorgioMarin, Catalin NicolaeBaccarelli, PaoloMarín, Josefa IsasiBade, AdityaMarin, MarinBaderna, DiegoMarinkovic, DraganBadshah, Mohsin AliMarinova, VeraBae, DowonMárk, Géza IstvánBae, Jong WookMarken, FrankBae, JoonhoMarkoutsa, EleniBae, WoorhamMarkowska-Szczupak, AgataBaev, AlexanderMarlow, FrankBaeza, AlejandroMarom, DanBaeza, MireiaMarotti De Sciarra, FrancescoBafekry, AsadollahMarques, Carlos Alberto FerreiraBahuguna, AshishMarques, Eduardo F.Baia, MonicaMarques, PaulaBaibarac, MihaelaMárquez, FranciscoBaig, NadeemMarradi, MarcoBaik, Sung-IlMarrazzo, Vincenzo RomanoBaimova, Julia A.Marszalek, MartaBaino, FrancescoMartin, Andrew ReadBajda, TomaszMartin, Inocencio R.Bakandritsos, AristeidisMartín, José IBaker, TharMartin, PascalBakina, Olga V.Martinelli, ChiaraBakkers, ErikMartinet, SébastienBakos, IstvanMartinez Rodrigo, JavierBaksi, AnanyaMartinez, VirginiaBakulin, AlexanderMartínez-Guitarte, José-luisBalamurugan, JayaramanMartínez-Huitle, Carlos A.Balandin, AlexanderMartínez-Lillo, JoséBalasoiu, MariaMartínez-Lostao, LuisBalaz, AntunMartinez-Martinez, Antonio J.Balazsi, CsabaMartinez-Martinez, DiegoBalcar, HynekMartínez-Martínez, VirginiaBald, IlkoMartínez-Pedrero, FernandoBaldino, LuciaMartinez-Triguero, JoaquinBaldo, NicolaMartin-Garcia, BeatrizBaldomir, DanielMartín-Gil, JesúsBaldoví Jachán, José JaimeMartini, ElisabettaBalema, ViktorMartino, MarcoBalen, BiljanaMartino, SabataBalestrazzi, AlmaMartins, José A.Balin, KatarzynaMartins, Rui C.Balint, ZsoltMartucci, AlessandroBall, ChristopherMartucciello, NadiaBalme, SébastienMarucho, MarceloBaltakys, KęstutisMaruyama, ShingoBalzer, FrankMarx, MichaelBan, IrenaMasař, MilanBan, YueMascia, LenoBanaprmath, N RMasciocchi, NorbertoBandyopadhyay, SmarakMashimo, TsutomuBanerjee, SantanuMasi, SofiaBanerjee, WritamMasino, MatteoBanerji, SourangsuMaslov, MikhailBanks, Craig E.Mason, JaradBannov, AlexanderMASROUR, RachidBanti, Christina N.Mastani Joybari, MahmoodBañuelos, JorgeMasud, JahangirBao, WenzhongMasuda, YoshitakeBarabanova, Anna I.Matei, CristianBarachevsky, Valery A.Matejíček, JiříBaranchikov, AlexanderMateo, Carmen ReyesBaranov, Andre NMaterer, Nicholas F.Baranović, GoranMathiyalagan, PrabhuBarbiellini, BernardoMatos, InêsBarbier, A.Matos, MariaBarbillon, GrégoryMatras-Postołek, KatarzynaBarbinta-Patrascu, Marcela-ElisabetaMatsoukas, ThemisBarbosa, Anderson L. R.Matsuda, IwaoBarbosa, PaulaMatsuda, ShoichiBarbuto, MirkoMatsumoto, Ken-IchiroBarek, JiriMattarelli, MaurizioBarille, RegisMatthes, RutgerBark, Chung WungMatthews, LorinBarkauskas, JurgisMatthews, PeterBarker, Philip JMattoscio, DomenicoBarlow, AndersMatwijczuk, ArkadiuszBaron, ByronMátyás, HunyadiBarone, CarloMatykiewicz, DanutaBarra, Guilherme M.O.Mauk, Michael G.Barranco, AngelMauriz, ElbaBarras, AlexandreMauro, MatteoBarreca, DavideMaxim, CătălinBarrera, G.Mayer, ThomasBarretta, RaffaeleMazeika, KestutisBarron, Ana Laura LunaMazierski, PawełBarsan, Madalina M.Mazur, UrsulaBarth, SvenMazurek, SylwesterBartkowiak, ArturMazurkiewicz-Pawlicka, MartaBartoli, MattiaMazyck, David W.Bartolomeo, Antonio DiMcCoy, Thomas M.Baryshnikov, Sergey VasilevichMcdonald, PeterBasak, Animesh KumarMcGlynn, EndaBaschieri, AndreaMcIlroy, David N.Baskoutas, SotiriosMcLeod, EuanBasoli, FrancescoMcManamon, PaulBassyouni, M. I.Męczyńska-Wielgosz, SylwiaBastos, Alexandre CunhaMeder, FabianBastos-Arrieta, JulioMedvecky, LubomirBastounis, EffieMedvidović-Kosanović, MartinaBatish, Daizy R.Meen, Teen-HangBatmunkh, MunkhbayarMegiel, ElżbietaBator, GrażynaMehl, GeorgBattiato, AlfioMehryan, Seyed Abdollah MansouriBattistel, AlbertoMei, BastianBauer, MarisMei, XuesongBauer, WolfgangMei, YunhuiBaumgartner, WernerMelero, RemediosBazlov, Andrey I.Mélinon, PatriceBazzan, MarcoMelinte, VioletaBear, Joseph C.Melle-Franco, ManuelBechelany, MikhaelMelnyk, InnaBecker, WilhelmMelo, AndréBecucci, MaurizioMeloni, EugenioBednarz, SzczepanMenaa, FaridBehrens, SilkeMenard, DavidBejan, IustinianMénard-Moyon, CéciliaBejtka, KatarzynaMeng, DongBekbölet, BekböletMeng, GuowenBekiari, VlasoulaMeng, HongBelardini, AlessandroMeng, XiangchaoBelcarz, AnnaMenger, Fredric M.Belenov, SergeyMenichetti, LucaBella, FedericoMenini, MariaBellani, SebastianoMensi, MounirBellmann-Sickert, KathrinMenzel, DirkBello, Nicholas T.Menzel, RobertBellos, EvangelosMenziani, Maria CristinaBellucci, AlessandroMerabia, SamyBelskaya, Olga B.Merano, MicheleBeltran, Ana M.Mercante, LuizaBelyakov, SergeyMere, ArvoBelykh, EvgeniiMereshchenko, AndreyBen Sedrine, NebihaMeroni, DanielaBender, PhilippMertens, ChristianBending, SimonMesaros, AmaliaBenedek, GiorgioMessina, Grazia Maria LuciaBenedetti, AlessioMessori, LuigiBenedetti, StefaniaMetson, James BernardBenedic, FabienMeulebroeck, WendyBenedikovic, DanielMeyer, JannikBenitez De La Torre, AlmudenaMeyyappan, M.Bensalah, NasrMezzetta, AndreaBenter, ThorstenMiao, QingqingBento, FatimaMican, SeverBeobide, Amaia SotoMichael, AdrianBerberan-Santos, MarioMichail, ChristosBerciu, MonaMichalcová, AlenaBerdonosov, PeterMichalkiewicz, BeataBerenguer-Murcia, ÁngelMichalska, MonikaBergamaschini, RobertoMicheletti, GabrieleBergdahl, AndreasMicheli, LauraBerisha, AvniMichl, ThomasBerkó, AndrásMichna, AnetaBerna, JoseMiculescu, FlorinBernardeschi, MargheritaMiecznikowski, KrzysztofBernardo, GabrielMielewczyk-Gryn, AleksandraBernardo, Luis Filipe AlmeidaMikhail, SoldatovBernascon, LeonardoMikhailov, Oleg V.Bernasconi, MarcoMikhlin, YuriBernatová, SilvieMiklaszewski, AndrzejBernhard, ChristianMikšová, RomanaBertaina, SylvainMikulics, MartinBerthod, PatriceMilanesio, MarcoBertolini, GiuliaMilani, PaoloBertolotti, MarioMilano, FrancescoBertoncello, PaoloMilasius, RimvydasBertrand, CzarnyMilcarek, ChristineBertsch, ArnaudMilella, AntonellaBertsch, PaulMiletto, IvanaBessa, Miguel A.Milewska, Maria J.Bessais, LotfiMilioto, StefanaBesser, MartinMilisav, IrinaBessergenev, Valentin G.Miljanić, SnežanaBesteiro, Lucas V.Milkova, ViktoriaBestetti, MassimilianoMiller, Ronald E.Betancourt, TaniaMilosevic, MiloradBettache, NadirMilowska, KarolinaBettati, StefanoMilyaev, MihkailBettencourt, Ana FranciscaMin Kyoon, ShinBettini, SimonaMin, XinBeuran, MirceaMinakshi, ManickamBezdetnaya-Bolotine, LinaMinamikawa, TakeoBhadra, Biswa NathMinarchick, ValerieBhandari, KhagendraMincheva, RosicaBhattacharya, SumitMinea, Alina AdrianaBhattacharyya, SanjibMinegishi, TsutomuBhatti, M.MMineo, PlacidoBhaumik, AnaghMiniewicz, AndrzejBhowmik, Pradip K.Minin, Igor V.Bhuvanesh, SrinivasanMino, LorenzoBi, SaiMintcheva, NeliBianchi, MassimilianoMinucci, SergioBianco, ArmandodorianoMiquelard-Garnier, GuillaumeBica, IoanMiqueu, ChristelleBidulská, JanaMirabella, Salvo Biesalski, MarkusMiranda, João MárioBietti, SergioMirante, FátimaBiezma, Maria VictoriaMiravet, Juan F.Bigham, AshkanMirkhani, Seyyed AlirezaBikiaris, DimitriosMiroslaw, BarbaraBikson, MaromMirvakili, Seyed MoBilal, SalmaMirzaei, AliBin, YaoMishakov, IlyaBini, FabianoMishra, NeerajBini, MarcellaMishra, Sanjay R.Bini, RobertoMishra, SuryakantBirdja, YuvrajMissell, FrankBirke, Kai PeterMistrik, JanBirke, Ronald L.Mitchell, AlecBirowosuto, Muhammad DanangMitchell, AndrewBisio, FrancescoMitchell, GeoffreyBisker, GiliMitchell, John S.Biswas, ChandanMititelu Tartau, LilianaBittencourt, CarlaMitomo, HideyukiBjorklund, RobertMitoraj, MariuszBlais, AnneMitropoulos, Athanasios C.Blaisten-Barojas, EstelaMittal, NiteshBlanco, IgnazioMittova, Irina YaBłasiak, SławomirMiura, YoshikoBlaszczuk, ArturMiyajima, YojiBlau, WernerMiyazaki, ToshikiBlaudeck, ThomasMiyoshi, MakotoBlayo, AnneMo, YiqunBludov, YuliyMoaca, Alina ElenaBlyakhman, Feliks AbramovichMocan, TeodoraBoanini, ElisaMocioiu, Oana CătălinaBobadilla, Luis F.Modanese, GiovanniBobitski, Yaroslav V.Modreanu, MirceaBoca, SandaMohamed, AbdulBocchetta, PatriziaMohammad, FaruqBodaghi, MahdiMohanram, HariniBoddapati, LoukyaMohapatra, Bidyut R.Bodzenta, JerzyMohles, VolkerBoehm, DanielaMoiseev, SergeyBogani, LapoMoissette, AlainBogdanowicz, RobertMoita, AnaBoghosian, SoghomonMokhov, Evgeniy N.Bohinc, KlemenMolas, MaciejBohm, SivasambuMolina, Carmen BelénBolotov, LeonidMolíns-Legua, C.Bonadies, IreneMolle, AlessandroBoncel, SlawomirMoloney, Mark G.Bondavalli, PaoloMombelli, Andrea W.Bonferoni, Maria CristinaMomekova, Denitsa B.Boni, Georgia AndraMonaghan, ScottBonomo, MatteoMonai, MatteoBonura, AngelaMondal, Amit KumarBonyár, AttilaMondal, GoutamBoomer, Jonathan S.Mondal, KunalBoothroyd, ChrisMondal, SubhadipBorchers, ChristineMondal, SudipBorhan, Adrian IulianMondeshki, MihailBorondics, FerencMondloch, JoeBoronin, Andrei I.Monguzzi, AngeloBorrega, MarcMonkawa, AkiraBorriello, AnnaMonobe, HirosatoBorsari, MarcoMontalenti, FrancescoBorst, Jan WillemMontanaro, LucioBose, Rajendran JCMontaño, Gabriel A.Bosnar, SanjaMonteil-Rivera, FannyBôta, AttilaMontemurro, MarcoBotelho, C MMontenegro, LuciaBotiz, IoanMontes, VicenteBottari, GiovanniMontessori, AndreaBotti, SabinaMontgomery, Jason M.Boudart, B.Monti, DonatoBoukha, ZouhairMonti, MarcoBoukos, Nikos K.Moon, Dae WoonBourel-Bonnet, LineMoon, Doo KyungBoutinguiza, MohamedMoon, Geon DaeBoybay, Muhammed SaidMoon, Kyoung-SeokBoyd, AnthonyMoore, Katie L.Boyer, SeverineMorais, Tânia S.Boytsova, OlgaMorallon, EmiliaBradac, CarloMordkovich, VladimirBraeuninger, PhilippMoreira, André FerreiraBraic, ViorelMorell, GerardoBraicu, CorneliaMorena, FrancescoBranda, FrancescoMoreno, CésarBrandbyge, MadsMoreno-Guzmán, MaríaBrandi, FernandoMorgado, JorgeBratek, ZoltánMorgan, Nicole Y.Bratek-Skicki, AnnaMorgunov, IgorBratsos, IoannisMori, WasukeBräu, LambertMorimoto, NobuyukiBrauch, UweMoritomo, YutakaBraveenth, RamanaskandaMorkoc, HadisBreidenstein, BerndMorozan, AdinaBreunig, Hans GeorgMorozov, Oleg G.Bridges, DenzelMorozov, Vladimir N.Britton, JonathanMorra, MarcoBroniatowski, MarcinMorris, Jeffrey F.Broquin, Jean-EmmanuelMortazavi, BohayraBrousse, ThierryMortimer, MonikaBrouzgou, ΑngelikiMosa, IslamBrunella, ValentinaMoscato, StefaniaBrunner, EikeMoseenkov, Sergey I.Bruno, ElenaMoshnyaga, VasilyBruno, LucaMoskovskikh, DmitryBrutti, SergioMott, Derrick M.Brylev, Oleg A.Motz, ChristianBrzhezinskaya, MariaMouchliadis, LeonidasBrzóska, KamilMoulick, AmitavaBu, LintaoMourdikoudis, StefanosBubnov, AlexeyMousa, Hamouda M.Buchet, ReneMousavi, Seyyed MojtabaBuckner, Steven W.Mousavi, ZekraBucur, BogdanMovahedi, NimaBuhl, Eva MiriamMoya, Antonio A.Buitenhuis, JohanMoya, CristianBuiu, OctavianMoya, JoséS SerafínBukreeva, TatianaMoya, SergioBulgariu, LauraMuhammad, SajjadBulusheva, LyubovMukai, YasuhitoBundaleska, NeliMukherjee, DibyenduBungau, SimonaMukherjee, SoumyaBuntinx, MiekeMukhin, IvanBuonomenna, Maria GiovannaMüller, ChristianBurakovsky, LeonidMüller, Thomas ErnstBurkhardt, UlrichMünchow, Eliseu AldrighiBurmistrov, V. A.Munir, KhurramBurokur, Shah NawazMuniz, André R.Bury, WojciechMuniz-Miranda, MaurizioBusby, YanMuñoz-Bonilla, AlexandraBusquets, Maria AntòniaMuñoz-Sandoval, EmilioBussolati, OvidioMunteanu, BogdanBusuioc, CristinaMunteanu, Florentina-DanielaBuszewski, BogusławMuraki, KatsuhikoButkute, RenataMuráth, SzabolcsButt, M. AliMuratov, DmitryButt, Muhammad AliMurcia-López, SebastiánBystrov, VladimirMurias, MarekCabaleiro, DavidMurthy, DharmapuraCaballero, AlvaroMuruganandan, ShanmugamCabanelas Valcárcel, Juan CarlosMurzina, Tatiana V.Cabanos, CerroneMuscio, AlbertoCabello, MartaMusic, DenisCabot, AndreuMusić, SvetozarCabral, HoracioMusmarra, DinoCabrales, LuisMussano, FedericoCabrele, ChiaraMustafina, AsiyaCabrera, HumbertoMustata, Fanica R.Cacciotti, IlariaMusuc, Adina MagdalenaCada, MartinMuter, Olga A.Cadar, OanaMuthaiah, ShellaiahCadatal-Raduban, MarilouMuzioł, Tadeusz M.Cadillon, LuisMuzykantov, Vladimir R.Cagnetta, GiovanniNa, Jun-HeeCai, GiampieroNa, KunCai, HongmeiNabeshima, FuyukiCai, JianchaoNabiałek, MarcinCaizer, CosticaNabil, BouaziziCalabrese, GiovannaNabiyouni, GholamrezaCalabria, DonatoNabok, AlexeiCalabrò, FrancescoNaccache, RafikCalamante, MassimoNada, Amr A.Calame, MichelNaeimirad, MohammadrezaCalatayud, David G.Nagai, HirokiCaldas, PauloNagai, MasayaCalero, Olga CaballeroNagase, TakeshiCalhelha, Ricardo CostaNagatani, NaokiCalifano, ValeriaNageswaran, ShubhaCĂLȚUN, Ovidiu FlorinNagy, EndreCalvaresi, MatteoNagy, Janos B.Calvo, Mauricio E.Najarian, SiamakCalzada, M. LourdesNajlah, MohammadCamargo, PedroNakamura, YoshiakiCambier, SébastienNakashima, NaotoshiCamblor, Miguel A.Nakashima, TakuyaCamerlingo, CarloNakayama, TomonobuCameron, DavidNakayama, YuushouCametti, CesareNakhchi, Mahdi ErfanianCamilli, LucaNalbach, PeterCamin, BettinaNam, InhoCaminade, Anne-MarieNam, MinwooCaminati, GabriellaNam, SeunghoonCamino, FernandoNam, Yun-SikCamino, GiovanniNan, AlexandrinaCammas-Marion, SandrineNan, HaiyanCampabadal, FrancescaNand, AshveenCampbell, Sawyer D.Nara, ShigetoshiCampos, ArmandoNarajczyk, MagdalenaCampos-Martínez, J.Narciso, JavierČanađija, MarkoNarojczyk, Jakub W.Canejo, João PauloNarra, SudhakarCanet-Ferrer, JosepNashchekin, Alexey V.Canetta, ElisabettaNasillo, GiorgioCannas, MarcoNasiri, NoushinCannavale, AlessandroNäslund, Lars-ÅkeCannistraro, SalvatoreNataf, Guillaume F.Cannone Falchetto, AugustoNatale, PaoloCano Luna, ManuelNatali Sora, IsabellaCao, DerongNaumov, AndreyCao, HongcuiNaumov, Anton V.Cao, JianyunNaushad, MuCao, MaoshengNavid, KashaninejadCao, MeiwenNaydenova, IzabellaCao, QiNazarchuk, EvgenyCao, ShuaiNazaruk, EwaCao, XudongNazemi, AliCao, YaoyuNeagu, AdrianCao, YiNechifor, GheorgheCao, YimingNeculita, Carmen MihaelaCapparè, PaoloNedelchev, LianCappellano, GiuseppeNeethirajan, SureshCappelletti, GiuseppeNegrutiu, MedaCappello, TizianaNehmé, ReineCaprara, SergioNeisiany, Rasoul EsmaeelyCăprărescu, SimonaNémeth, PéterCaprio, Fabrizio DiNemnes, George AlexandruCapsoni, DorettaNenning, AndreasCarabineiro, SoniaNesterov, DmytroCaramori, StefanoNesterova, Oksana V.Carbajo, JaimeNeugebauer, WitoldCarbone, MarilenaNeumann, DetlefCardoso, ClaudiaNeumann, SebastianCargnoni, FaustoNeupane, KrishnaCaridad, JoseNeverov, Vladimir N.Carlino, ElvioNeves, Ana M.A.Carlucci, ClaudiaNeves, Cristina Sofia Dos SantosCarmona, FranciscoNevshupa, Roman A.Carmona, ManuelNewbury, Dale E.Carniato, FabioNewton, Graham N.Caro, CarlosNezamzadeh-Ejhieh, AlirezaCarradori, SimoneNguyen, Khoi TanCarravetta, VincenzoNguyen, Ngoc A.Carreau, PierreNguyen, Nhat TruongCarrete Montaña, JesúsNguyen, Nhung H. A.Carter, KatharineNguyen, Van-HuyCaruntu, ConstantinNi, JiangfengCaruntu, DanielaNiaura, GediminasCaruntu, GabrielNica, PetruCaruso, GerardoNicholson, DavidCaruso, UgoNicoletta, Fiore PasqualeCasado-Coterillo, ClaraNiculescu-Duvaz, DanCasarin, MaurizioNie, LimingCasassa, SilviaNie, YuanCaschera, DanielaNieckarz, DamianCasella, PatriziaNiedziela, TomaszCaserta, SergioNiemirowicz-Laskowska, KatarzynaCasida, MarkNievergelt, Adrian PascalCassagnau, PhilippeNikolaidis, Alexandros K.Cassidy, John F.Nikolakakis, IoannisCastaldi, GiuseppeNikolay V., SidorovCastaldo, RacheleNikumb, SuwasCastán, HelenaNilsson, FritjofCastell, PereNing, JiqiangCastellanos-Gomez, AndresNisbet, AndrewCastelletto, StefaniaNishiyama, YoshioCastelli, Ivano E.Nisnevitch, MarinaCastellino, MicaelaNissinen, MaijaCastilla, Ana M.Nistor, Ileana DenisaCastro-Muñoz, RobertoNitodas, Stephanos F.Casula, MicheleNitta, JunsakuCataldi, AmeliaNiu, ShichaoCavaliere, ChiaraNiu, XianghengCavaliere, EmanueleNIwa, OsamuCavalleri, OrnellaNobre, Marcos Augusto LimaCavallini, MassimilianoNocca, GiuseppinaCavigli, LuciaNocchetti, MorenaCaviglioli, GabrieleNoel, Jean-MarcCazan, CristinaNoël, SébastienCebecauer, MarekNoetzel, RichardCebrián Guajardo, SusanaNogales, AuroraCecilia, HodurNogales, EmilioCedervall, JohanNoh, HeesoCefalas, Alciviadis-ConstantinosNoirez, LaurenceCelichowski, GrzegorzNoma, NaokiCempura, GrzegorzNomura, ShintaroCenian, AdamNonappa, NonappaCenteno, Miguel AngelNonnenmann, Stephen S.Cepeda, JavierNoor, Noor Fadiya MohdCerdan, LuisNorek, MałgorzataCeresoli, DavideNorton, MichaelCernea, MarinNotas, GeorgeCerulli, R.Noubactep, ChicgouaCervellati, FrancoNouvel, CécileCervelló, Gemma GutiérrezNovikov, Alexander S.Ceulemans, ArnoutNovio, FernandoCha, ChaenyungNovitsky, AndreyChaban, Vitaly V.Novotny, ZbynekChachkov, Denis V.Nowaczyk, JacekChae, SujongNowak, AdrianaChakraborty, DebayanNowak, PawelChakraborty, IndranathNowicka, Anna MariaChaldyshev, VladimirNowicki, MarekChalioris, ConstantinNugroho, Ferry Anggoro ArdyChamberlin, RalphNunes, Cláudia M.Chandraiahgari, Chandrakanth ReddyNuñez, RosarioChandrasekhar, ArunkumarNunzi, FrancescaChang, Chia-ChenNyushkov, Boris N.Chang, Chih-WeiObraztsov, Alexander N.Chang, Ching-JinO’Brien, Paul G.Chang, Hai-ChouObrzut, JanChang, Hye JungOchiai, BungoChang, Hyo SikOćwieja, MagdalenaChang, Jia-YawOda, TakujiChang, Long-SenOdegard, GregoryChang, Sheng-PoOdent, JérémyChang, SheryOehler, FabriceChang, Wen-HueiOezkan, SeldaChantana, JakapanOgawa, ShinpeiChappel, EricOgawa, ToshioCharles Andrew, DowningOguey, ChristopheCharles, Andrew D. M.Ohashi, MasashiChatterjee, SandipOhisa, SatoruChatterjee, SurajitOhishi, YujiChatzimitakos, Theodoros G.Ohmi, Shun-ichiroChauvin, NicolasOhno, SeigoChava, Rama KrishnaOhsawa, TakeoChavez-Angel, EmigdioOhto, KeisukeChe, RenchaoOka, KengoChellappan, Dinesh KumarOkada, NarihitoChen, BingdiOkajima, JunnosukeChen, BoOkamoto, MasamiChen, ChiachungOkamoto, YoshiharuChen, Chia-YunOkazaki, JojiChen, Chih-KuangOkazaki, ToshiyaChen, Chih-YenOkhay, Olena I.Chen, Chin-YiOksman, KristiinaChen, DaiqinOkulov, IlyaChen, DazhengOlaizola, Santiago M.Chen, DongOlar, RodicaChen, DongchengOlenin, Andrei YuChen, Fang-ChungOlewnik-Kruszkowska, EwaChen, FulinOliveira, João PedroChen, GuanyingOliveira, MonicaChen, GuohaiOliveira, SaraChen, HongleiOliver, Allen G.Chen, HsiangOliver-Meseguer, JuditChen, HuiyuOlsen, Lars FolkeChen, I-Wen PeterOms-Oliu, GemmaChen, Jhy-DerOnaka, SusumuChen, JianjunOndicˇ, LukášChen, Jian-ZhangOnodera, TakashiChen, JihuaOnwudiwe, DamianChen, JunOpitz, JörgChen, KeOpra, DenisChen, Kelvin H.-C.Oprea, AlexandruChen, Kuan-RenOprea, OvidiuChen, Liang-YuO’Rear, EdgarChen, LingenOreshonkov, AleksandrChen, Lung-ChienOrge, CarlaChen, QixianOrian, LauraChen, QuarkOrlandi, MicheleChen, RongOrlovskii, YuriiChen, San-YuanOrłowska, B. LesiakChen, Sheng-ChiOrmianer, ZeevChen, ShihuaOrtega, José MarcosChen, Tao-HsingOrtelli, SimonaChen, Tei-ChenOrtix, CarmineChen, WanpingOrtiz, DoloresChen, Wan-TingOrtore, Maria GraziaChen, WeiOsakada, YasukoChen, Wen-ChengOsaki, TomohiroChen, WenwenOschatz, MartinChen, XiangzhongOsella, Alberto RubénChen, XianpingOsella, SilvioChen, XuechengOshikiri, TomoyaChen, XuweiOsipov, AndreyChen, Yen-HwaOsipov, VladimirChen, YuOthman, AliChen, Yu-ChangOtosu, TakuhiroCHEN, YU-CHENGOtřísal, PavelChen, YuncongOu, QingdongChen, Yun-ShengOuellet-Plamondon, ClaudianeChen, Zhe-shengOuyang, Xiao-kunChen, ZhileiOuyang, ZhengbiaoChen, ZhizhongOya, TakahideChen, ZhongningOzawa, YoshikiChenevier, PascaleOzturk, Mehmet C.Cheng, Chih-ChiaPablos, CristinaCheng, Chung-WeiPace, BernardoCheng, Fong-YuPachapur, VinayakCheng, GangPachfule, PradipCheng, GuanghuaPaczesny, JanCheng, Huanyu (Larry)Padmanabhan, Sanosh Kunjalukkal PadmanabhanCheng, I.Padnya, PavelCheng, JipengPadrão, JorgeCheng, JipingPagano, StefanoCheng, QilinPaineau, ErwanCheng, XiaopengPaizs, CsabaCheng, Yang-TsePak, SangyeonCheng, ZhenzhouPak, SoojongCheraghian, GoshtaspPakharukova, Vera P.Chernyak, SergeiPalacios, Juan JoseChernyshev, VladimirPalau, AnnaCherukula, KondareddyPall, EmokeChetcuti, Michael J.Pallavicini, PiersandroChiang, Hai-PangPallecchi, IlariaChiang, KenPalleschi, VincenzoChiang, Long YPalma-Dibb, Regina GuenkaChiang, Ming-HsiPalmieri, ValentinaChiang, Wei-HungPaloncýová, MarkétaChiappini, AndreaPalumbo, FabioChieh, Jen-JiePalumbo, GaetanoChien, Hsiu-WenPamies, RamonChigo-Anota, ErnestoPan, GangChinappi, MauroPan, Guan-TingChiper, ManuelaPan, JianChiriac, Aurica P.Pan, JianmingChiriac, HoriaPan, LikunChirico, GiuseppePan, Ye-TangChirumamilla, ManoharPan, ZhuChirvony, VladimirPanagopoulos, C. N.Chis, VasilePanchal, SatyamChitgupi, UpendraPancielejko, MieczysławChiu, Fang-ChyouPanczyk, TomaszChiu, Nan-FuPandey, A.K.Chiu, Tai-ChiaPandey, PuranChiu, Te-WeiPandey, SadanandChizhik, Alexey I.Panek, PiotrChmielarz, LucjanPaneth, PiotrChmielarz, PawełPang, HuanChmielowska-Bąk, JagnaPang, XiaoluCho, ByungjinPang, YoonsooCho, Cheong-WeonPanin, SergeyCho, Eun JeongPankratov, VladimirCho, GuyoungPanneerselvam, RajapandiyanCho, Hyun-JongPanniello, AnnamariaCho, In SunPanomsuwan, GasiditCho, Jung SangPanos, PapagiannakopoulosCho, Jung YoungPant, BishweshwarCho, Man HoPantano, PaulChoi, ByungchulPanzarasa, GuidoChoi, HongseokPanzarini, ElisaChoi, Jane RuPaolella, AndreaChoi, Jeong RyeolPaolicelli, GuidoChoi, Jong-ryulPaolone, AnnalisaChoi, JungwookPap, József SándorChoi, KeunsuPap, SabolcChoi, Myong YongPapa, FloricaChoi, Nag JungPapadogiannis, NektariosChoi, Seok KiPapadopoulos, ChrisChoi, Yong ChanPapakonstantinou, Christos G.Choi, Yong GyuPapalazarou, EvangelosChoi, YongManPapanikolaou, NikolaosChojnowski, JulianPapathanassiou, Anthony N.Chollet, Jean-FrançoisPapatriantafyllopoulou, ConstantinaChoo, JaebumPapoulis, DimitriosChoopun, SupabPappas, DimitriChorazy, SzymonPappritz, KathleenChou, Shih-FengPaquette, BenoitChow, Franklin Wang-NgaiPar, MatejChow, James C. L.Parajuli, DurgaChowdhury, Ezharul HoqueParale, Vinayak G.Chrcanovic, BrunoPardo, JoséChrissafis, KonstantinosParisi, FilippoChristensen, Jørn BPark, Byung-WookChristoforidis, KonstantinosPark, Chul-hoChristol, PhilippePark, Deok-YongChristov, Ivan P.Park, Eun DuckChrobak, ArturPark, Helen HejinChroni, AngelikiPark, Jeong YoungChu, Chen-ShanePark, JongsikChu, Po-ChenPark, Joo-IlChu, ZhiqinPark, JunsooChuah, Chong YangPark, JunyongChuang, Andrew (Chihpin)Park, Kang HyunChuang, ChiashainPark, Ki SooChuang, Hung-YiPark, KibogChuang, Yen-JunPark, Kwang-hyunChung, KunookPark, Kyu ChangChung, Ren-JeiPark, Min BumChung, Sheng-HengPark, Myoung-HwanChusuei, CharlesPark, NokukChuvil’deev, V. N.Park, Sang JoonChwastek, KrzysztofPark, Seon JooCiampi, SimonePark, Seung-ChunCiani, GiovanniPark, Shin-HyungCiapetti, GabrielaPark, SudongCicogna, FrancescaPark, SunghaCid-Samamed, AntonioPark, SungjuneCieśliński, JanuszPark, Yong TaeCimpoesu, NicanorPark, YuriCinelli, PatriziaParlapiano, IsabellaCinteza, OtiliaParlinska-Wojtan, MagdalenaCioffi, NicolaParmeggiani, CamillaCiolaciu, DianaParra, RubenCipak Gasparovic, AnaPasán, JorgeCirillo, GiuseppePascual, Juan PabloCirlin, GeorgePascual, LauraCirujano, Francisco G.Pascuta, PetruCivalek, ÖmerPashkin, OleksiyClarke, RonPasinszki, TiborClegg, Jack K.Paskaleva, AlbenaClement, JoachimPasqua, LuigiClement, YuenPasquato, LuciaCobianu, CornelPasquinelli, GianandreaCochis, AndreaPassante, RobertoCoclite, Anna MariaPassero, Luiz Felipe DominguesCoduri, MauroPassow, ThorstenCoelho, LuisPastero, LindaCoenen, Jan WillemPastore, CarloCohn, Robert W.Pasuk, IulianaCoisson, MarcoPatanè, SalvatoreCojocaru, CorneliuPatel, KapilCojocaru-Mirédin, OanaPaternò, Giuseppe MariaColla, ValentinaPaterno, Leonardo GiordanoCollar, Emilia P.Pathak, RajeshCollings, NeilPatil, Swati J.Collini, MaddalenaPato Doldan, BreoganColombani, JeanPatolsky, FernandoColombo, ChiaraPatra, Jayanta KumarColombo, GiorgioPatra, NiranjanColombo, Luigi Pietro MariaPatroi, DeliaColomer, Jean-FrancoisPatti, AlessandroColumbu, StefanoPatzelt, AlexaComan, CristinaPaul, RajibComan, VasilePauliac-Vaujour, EmmanuelleComanescu, CezarPauliukaite, RasaComes-Franchini, MauroPaulusse, JosComfort, Kristen K.Pavel, NicolaieConcellón, AlbertoPavel, Octavian DumitruConcetta Valeria Lucia, GiosafattoPavlík, ZbyšekCondorelli, Guglielmo GuidoPawlak, MichałCong, LongqingPeale, RobertConnolly, James P.Peana, Massimiliano F.Consoli, Grazia M.l.Pecz, BelaConstantin, LucianPeer, PetraConstantinescu, Cătălin-DanielPeera, Shaik GouseContaldo, MariaPei, ZingwayConzuelo, FelipePeighambardoust, Seyed HadiCook, Matthew C.Peiner, ErwinČopič, MartinPeixinho, JorgeCopolovici, Dana MariaPeixoto De Almeida, MiguelCoppola, AntonioPekař, MiloslavCoppola, SaraPeljo, PekkaCordani, MarcoPellegrino, PaoloCórdoba, ManuelPelli, StefanoCórdoba, RosaPellicer, EvaCormack, Alastair N.Peng, ChangCormia, Robert D.Peng, CuiCorpino, RiccardoPeng, FeiCorregidor, VictoriaPeng, JunbiaoCorreia, Ilídio JPeng, XiangheCorsaro, CarmeloPeng, XiaoniuCorsi, IlariaPensa, EvangelinaCortaberria, GalderPeppel, TimCortese, BarbaraPerale, GiuseppeCortés-Hernández, D. A.Perche, FedericoCorti, Horacio R.Perduca, MassimilianoCoscia, Maria RosariaPereira Gonçalves, AntónioCosco, DonatoPereira, EulaliaCosma, PinalysaPereira, Eulalia CarvalhoCosta, Carlos MiguelPereira, Fernando J.Costa, DianaPereira, LucianaCosta, Manuel Filipe Pereira Cunha MartinsPereira, Luís M.N.P.Costa, Rui S.Pereira, LuizCouce, María D.Pereira, Maria CarmoCouque, HervéPereiro, JuanCoy, EmersonPereiro, RosarioCraciun, Ana-MariaPerero, SergioCragg, PeterPeres, António M.Credico, Barbara DiPerevedentseva, ElenaCrespi, AndreaPerevyazko, IgorCrisp, Ryan W.Pérez -Coll, D.Cristea, CeciliaPerez Del Pino, AngelCristea, DanPérez García, LucasCristescu, RodicaPérez Puyana, Víctor ManuelCristian, PetcuPérez, AntonioCristóbal, JuliaPérez, ErnestoCroce, Anna CletaPérez-Prieto, JuliaCrunteanu, AurelianPergher, Sibele B.C.Cruz, SandraPergolesi, DanieleCsapo, EditPerinelli, Diego RomanoCsapó, EditPerioli, LuanaCsík, GabriellaPeris, José-EstebanCtistis, GeorgiosPerisanu, SorinCuesta, RafaelPerna, GiuseppeCui, BoPerov, NikolaiCui, JianleiPerova, Tatiana S.Cui, WanzhaoPerrin, LaraCulita, Daniela CristinaPersichetti, LucaCurran, SeamusPerteghella, SaraCurulli, AntonellaPerucchi, AndreaCutler, Christopher WPerumal, SugunaCuypers, DieterPessoa, Rodrigo SávioCzeppe, TomaszPetala, AthanasiaCzogalla, AleksanderPetcu, CristianD’ Acunto, MarioPetelska, AnetaDa Costa Ribeiro, Ana PaulaPeter, AncaDa Costa, Ana Maria Dos Santos RosaPeter, LaszloDa Silva, Luís PintoPéter, LaszloDa Silva, Pedro RaposeiroPetersen, ElijahDabrowski, JanuszPeterson, G.P.Dacarro, GiacomoPetit, ChristopheDagmara, MalinaPetralia, SalvatoreD’Agostino, CarminePetralito, StefaniaDai, NingPetridis, CostasDai, ShengPetrov, DmitryDai, YejingPetrov, MihailDai, ZhigaoPetrovykh, Dmitri YDaiguebonne, CarolePetruczynik, AnnaD’Alfonso, LauraPetry, WinfriedDalhaimer, PaulPetukhov, Andrei V.Dallos, AndrásPeukert, WolfgangDalmo, RoyPeviani, MarcoDalmoro, AnnalisaPezeshki, AtiyeD’Amico, FrancescoPfnür, HerbertDAMIN, Alessandro AliPham, Hong DucDamma, DevaiahPham, Xuan-HungDamodaran, KrishnanPhan, Manh-HuongD’Amore, MarcelloPhilipose, UshaD’andrea, CristianoPiano, IlariaDang, AleiPiattelli, AdrianoD’Angelo, GiovannaPicca, Rosaria AnnaDaniele, StéphanePiccialli, GennaroDanilson, MatiPiccirillo, ClaraDanko, MartinPichon, LaurentD’Anna, FrancescaPichon, LionelDantelle, GéraldinePiecek, WiktorDanti, SerenaPiedade, Ana PaulaDao, Van-DuongPielichowska, KingaD’Archivio, Angelo AntonioPielichowski, KrzysztofDarling, SethPiliougine, MichelDarmanin, ThierryPilot, RobertoDas, BhaskarPina, Maria PilarDas, JagotamoyPina, SandraDas, Narayan ChandraPinčák, RichardDas, PoushaliPineda Rodríguez, TeresaDas, RupaliPiñeiro, MartaDas, SubhaPiñero, Jose CarlosDashjav, EnkhtsetsegPingitore, NicholasDastan, DavoudPinson, JeanD’Auria, FrancescoPinteala, MarianaDavarpanah, AfshinPinter, GeraldDavid, ChristinPinto, ArturDavid, Gabriela IuliaPinto, Joana VazDavid, Iulia GabrielaPinto, SaraDavis, FredPIPISKA, MartinDavis, Ryan D.Pipiška, MartinDawes, JudithPircalabioru, Gratiela GradisteanuDe Almeida, Jose ManuelPisarcik, MartinDe Andrés, AliciaPiscioneri, AntonellaDe Angelis, CostantinoPisk, JanaDe Bonis, AngelaPiskunovs, SergejsDe Caro, DominiquePispas, StergiosDe Caro, VivianaPiszczek, PiotrDe Castro, Carlos A. NietoPiszczyk, ŁukaszDe Crescenzi, MaurizioPiticescu, Radu RobertDe Falco, GianluigiPitto-Barry, AnaïsDe Felipe Mesquida, DavidPivac, BrankoDe France, KevinPizzimenti, StefaniaDe Graaf, CoenPlachetka, UlrichDe La Fuente, Germán F.Placke, TobiasDe La Fuente, RaúlPlain, JérômeDe La Presa, PatriciaPleixats, RoserDe Leo, NatasciaPlewa, JulianDe Luca, GiovannaPlumer, MartinDe Luca, PierantonioPlutino, Maria RosariaDe Luna-Bertos, ElviraPlyusnin, Victor F.De Marco, LuisaPochard, IsabelleDe Matteis, Fabio Podemski, PawełDe Matteis, ValeriaPodgursky, VitaliDe Melo, Marcelo M.r.Poe, BrentDe Oliveira Patrício, Patrícia Santiago O.Pogăcean, FlorinaDe Padova, PaolaPoggi, GiovannaDe Sio, LucianoPohl, PawelDe Spirito, MarcoPokrant, SimoneDe Stefano, LucaPokutnyǐ, Sergey I.De Teresa, Jose-MariaPolimeno, AntoninoDe Tommasi, EdoardoPolini, RiccardoDe Zotti, MartaPolito, LauraDebucquoy, MaartenPoljak, MirkoDeck, PaulPolop Jordá, CeliaDeguchi, ShigeruPolosan, SilviuDeimede, ValadoulaPoma, AnnaDejneka, AlexandrPomastowski, PawełDekhtyar, JurijsPonomarev, Igor I.Del Amo, VicentePons, JosefinaDel Buono, DanielePons, ThomasDel Hierro, IsabelPonseca, CarlitoDel Turco, SerenaPonterio, Rosina CelesteDelfanazari, KavehPonti, AlessandroDelfino, InesPoodt, PaulDelgado, Ángel V.Pop, ViorelDelgado-Aguilar, MarcPopa, AlexandruDella Pergola, RobertoPopa, LacramioaraDella Porta, GiovannaPopat, AmiraliDell’Agli, GianfrancoPopelka, AntonDell’Aglio, Marcella.Popescu, AndreiDell’Anna, RossanaPopescu, VasilicaDell’Isola, FrancescoPopielarz, RomanDell’Olio, FrancescoPopko, Ewa.Delogu, FrancescoPopkov, Vadim IgorevichDelongchamp, DeanPopov, AnatoliDelville, Marie-HelenePopov, MikhailDemel, JanPopova, MargaritaDemers, HendrixPorotnikova, NataliaDemetrescu, IoanaPortesi, ChiaraDeng, Hai-QiangPoŝa, MihaljDeng, LianwenPosselt, DortheDeng, Ming JayPostigo, Pablo AitorDeng, ZilanPostnikov, Pavel S.Deniaud, AurélienPoston, BrachDenizot, BenoitPotapov, AndreiDepla, DiederikPotdevin, AudreyDercz, GrzegorzPotrich, CristinaDeretzis, IoannisPoumellec, B.Derkowska-Zielinska, BeataPourrezaei, KambizDeschaume, OlivierPoyet, Jean-LucDesgranges, LionelPrado-Gonjal, JesúsDespotović, VesnaPrado-Gotor, RafaelDEVEL, MichelPrajzler, VaclavDevidson, SergioPramanik, ArindamDewapriya, NuwanPramanik, SubhamayDewil, RafPran, Elise RundénDézsi, LászlóPranckutė, RamintaDhar, GouravPrasad, AnkushDhiman, InduPrashanth, Konda GokuldossDi Battista, DavidePrat, MariaDi Corato, RiccardoPraus, PetrDi Gioia, Maria LuisaPravica, Michael G.Di Giovannantonio, MarcoPreda, NicoletaDi Liberto, GiovanniPredoi, DanielaDi Liddo, RosaPresto, SabrinaDi Marco, AlessandroPribyl, JanDi Maro, AntimoPricl, SabrinaDi Michele, AlessandroPrida, Victor M.Di Nardo, FabioPrieto, CristinaDi Palma, LucaPrieto, Miguel A.Di Pierro, ProsperoPrimc, GregorDi Pietro, PaolaPrimožič, Mateja J.Di Schino, AndreaPrincipe, MariaDiaconu, Bogdan M.Prior, JoãoDiaferia, CarloProchazka, IvanDiao, DongfengProchazka, MarekDíaz, EsperanzaProchowicz, DanielDíaz, EvaPron, AdamDiaz, Jose ManuelPronin, ArtemDíaz-Ortiz, ÁngelProsa, MarioDickey, MichaelProskurnin, MikhailDíez-Pascual, Ana MaríaProsviryakov, AlexeyDigiacomo, LucaProtesescu, LoredanaDilley, N RProux, OlivierDimakis, EmmanouilPrucnal, SlawomirDimitri, RossanaPrudkovskiy, Vladimir S.Dimitrievska, MirjanaPruna, AlinaDimitrov, IvayloPruna, Alina IulianaDimos, KonstantinosPruneanu, Stela-MariaDimov, NikolayPryamikov, AndreyDina, Nicoleta ElenaPtaszek, PawełDinca, ValentinaPu, MingboDinescu, SorinaPu, Ying-ChihDing, AnPuente-Santiago, AlainDing, BingPuerto, DanielDing, DongyanPuerto, Rodrigo AlcántaraDing, FeiPugachev, Alexey M.Ding, GuqiaoPuglia, DeboraDing, XinPuglisi, AlessandraDing, YichunPuglisi, RosariaDing, ZhaoPuigdollers, JoaquimDinh, ToanPujals, SílviaDinis, MárioPujol, MontserratDinischiotu, AncaPulgar, VictorDinu, Cerasela ZoicaPulham, ColinDionisio, GiuseppePuli, Venkata SreenivasDippong, ThomasPullman, David P.Ditaranto, NicolettaPum, DietmarDiţu, Lia MaraPurcar, VioletaDivitini, GiorgioPurcell-Milton, FinnDjeffal, F.Pussi, KatariinaDjellabi, RidhaPuszkiel, Julián A.Djourelov, NikolayPutkonen, Matti Il-mariDo, Trong-OnPuttisong, YuttapoomDobrzynski, PiotrPutz, Ana MariaDodero, AndreaPuvača, NikolaDodi, GianinaQasim, Syed Saad BDoganis, PhilipQi, CongDolado, Jorge S.Qi, HongDolganov, Pavel VladimirovichQi, KezhengDoll, KlausQi, PengDomashevskaya, Evelina P.Qi, ShengliDombrovsky, LeonidQi, XiaoliangDomenici, ValentinaQi, XueqiangDominguez-Alfaro, AntonioQian, DongDong, BoweiQian, KunDong, GuangnengQibin, LiDong, HaifengQin, DonghuanDong, JunhangQin, YuyueDong, WeiQiu, FengDong, XinglongQiu, KeqiangDong, ZhaogangQiu, LinDongil, Ana BelénQiu, WeiDonnadio, AnnaQuan, BinDonnini, SandraQuapp, WolfgangD’Onofrio, MariapinaQuarta, AlessandraD’Orazio, AntonellaQuesada, AdriánD’Orazio, FrancoQuynh Hoa, LeDorcioman, GabrielaRab, AndrásDorenbos, GertRabczuk, TimonDoris, EricRabiee, NavidDorozhkin, Sergey V.Rabilloud, FranckDorval Courchesne, Noémie-ManuelleRabin, YitzhakDos Santos, Elisama VieiraRabkin, EugenDostovalov, Alexander VladimirovichRachwalski, MichałDou, MeilingRack, Philip D.Doustkhah Heragh, EsmaeilRaczkowska, JoannaDoustkhah, EsmailRadacsi, NorbertDoutch, JamesRadamson, Henry HDouthwaite, MarkRadnik, J.Douvalis, Alexios P.Radtke, AleksandraDouvris, ChrisRadu, AndreeaDouzi, BadreddineRadu, Gabriel LucianDowney, AustinRadwański, KrzysztofDowon, BaeRadziuk, DaryaDrabowicz, JózefRafailov, Peter MetodievDragoman, DanielaRafatullah, MohdDragoman, MirceaRagab, TarekDrazic, GoranRaganati, FedericaDražić, GoranRagusa, AndreaDressel, MartinRahman, Md MahbuburDrillet, Jean-FrançoisRahman, Mohammad MizanurDrozdov, Aleksey D.Rahman, Muhammad KalimurDrozdov, AndreyRai, AkhileshDryzek, EwaRaikher, Yuriy L.Du, ChunfangRailean-Plugaru, VioricaDu, GuopingRaimondo, MarialuigiaDu, XuRainey, JanDu, XushengRajaji, UmamaheswariDu, ZhiyanRajput, VishnuDuan, LeipingRamadoss, AnanthakumarDuan, YanRamakrishnan, SayanthanDuay, JonathonRamallo, Manuel V.Dubrovskii, VladimirRamani, AlwarDudek, MirosławRamirez-Castellanos, JulioDudziec, BeataRamis, GianguidoDueñas Carazo, SalvadorRamos, DanielDuffy, RayRamos, JavierDuguet, EtienneRamos, Rocio LitranDulf, Eva H.Ramsköld, DanielDulova, NiinaRana, VikasDulski, MateuszRanc, VáclavDumitrica, TraianRanieri, AntonioDumitru, FlorinaRao, AshitDumur, FrédéricRao, RahulDunaevskiy, MikhailRapa, MariaDunbar, AlanRaposo, MariaDupin, DamienRaptis, IoannisDurach, MaximRäsänen, EsaDuraes, JoaoRasras, MahmoudDurazzo, AlessandraRasteiro, Maria GraçaDuta, LiviuRastin, HadiDutkiewicz, MichałRastogi, AlokDutta, SumanRatautaite, VilmaDvurechenskii, AnatoliyRathod, Vivek T.Dyachkov, PavelRatso, SanderDyakonov, G. S.Rau, IleanaDzantiev, B. B.Raucci, Maria GraziaDzhardimalieva, GulzhianRauscher, HubertDziedzic, AndrzejRauwel, ErwanDziembaj, RomanRauwel, ProtimaDzyuba, SergeiRavaine, SergeE. Golub, IgorRavelli, DavideE Serda, RitaRavera, EnricoÉber, NândorRavindra, Nuggehalli M.Ebralidze, Iraklii I.Ravindranadh, KoutavarapuEconomopoulos, Solon P.Ray, AniruddhaEdee, KofiRbah-Vidal, LatifaEdelman, Irina S.Read, JeffreyEdirisinghe, MohanRebezov, MaksimEdwards, JoshuaRebholz, ClausEdwards, Paul R.Rebollar, EstherEersels, KasperRebrov, EvgenyEfimov, AlexanderReddy, Govardhan N.ManjunathaEgberts, PhilipReddy, M. Siva PratapEglitis, RobertsReddy, M. V.Egorkin, VladimirReggiani, SusannaEgorov, Alexander V.Reguera, EdilsoEhrmann, AndreaReguero, MarEid, Basma M.Rehman, SarishEilmes, AndrzejReichhardt, CharlesEimutis, JuzeliūnasReid, Michael S.Ek Rosén, MartinReifsnider, Kenneth L.Ekimov, Evgeny AReinosa, Julián JiménezEl Marssi, Mimoun E.L.Reis, ANA TERESAEl Yamani, NaoualeRej, SouravElaissari, AbdelhamidRejado, Rodolfo CuernoElbendary, AshrafRella, RobertoEleni, MakaronaRemedios Serrano, DoloresEleuch, HichemRemes, ZdenekElguero, JoséRemsen, Edward E.Eligiusz, PostekRen, BiaoEliseeva, Svetlana V.Ren, NaElkady, MarwaRen, QinlongEl-Kemary, Maged AbdeltawabRen, YukunEllahi, RahmatRenu, SankarEllegaard, MarianneRepän, TaaviEllendt, NilsRestivo, JoãoEllinas, KosmasReuter, KlausElm, Matthias T.Reuther, JamesElmas, SaitReverchon, ErnestoElmquist, Randolph E.Rey, AnaElshafie, Hazem S.Reyes-Ortega, FelisaEl-Shishtawy, RedaReza, Khan MamunEltaher, MohamedRhee, Jong IlElviri, LisaRhiger, DavidElwakeel, KhalidRiassetto, DavidEl-Zohry, Ahmed M.Ribeiro, AlexandraEmelyanov, Andrey V.Ribeiro, Maria FilipaEmiliano, MeaurioRicart, SusagnaEmmanuel, LhuillierRiccardi, ClaudiaEncinas, ArmandoRicci, MarilenaEnculescu, IonutRiccio, RaffaeleEnesca, AlexandruRichard, Caroline S.Engel, JonathanRichard, CyrilleEngelmann, PeterRichards, ChrisEnglish, NiallRichter, GuntherEnikeev, Nariman A.Richter, Günther H.S.Ennen, IngaRichtera, LukášEnrichi, FrancescoRico-Gavira, VíctorEnyashin, AndreyRider, Andrew N.Eon, DavidRiedl, HelmutEpifani, MauroRiha, PavelEremeyev, Victor A.Riha, ShannonEric, TillmanRijckaert, HannesEriksen, René LyngeRimola, AlbertEriksson, JensRimpelová, SilvieEriksson, MirvaRinaldi, DanieleEritja, RamonRinaldi, RosariaESCAMILLA, ALFONSORinkevich, AnatolyEscobedo, CarlosRinnerthaler, MarkEscola, José MaríaRivadeneyra, AlmudenaEscorihuela, JorgeRivera-López, F.Escudero, CarlosRivero-Müller, AdolfoEsen, CemalRivoira, LucaEsenaliev, Rinat O.Rizal, ConradEspinoza-González, RodrigoRizzi, Gian AndreaEsposito, MarcoRobert, NewcombEspro, ClaudiaRoberto Giannuzzi, RobertoEspuelas, SocorroRobichaud, DavidEstellé, PatriceRocco, DavideEsteves Da Silva, Joaquim C.G.Rocco, LuciaEstrader, MartaRochat, SebastienEstrany, FrancescRochefort, Gael Y.Eubank, TimothyRodembusch, Fabiano S.Eugenio, FazioRodenbücher, ChristianEvangelisti, ClaudioRöder, StefanEvangelopoulos, MichaelRodil Rodríguez, EvaEvans, DrewRodionov, IlyaEvans, JulianRodionova, ValeriaEvlashin, Stanislav A.Rodrigues, DarioEvtukh, AnatoliyRodrigues, Lucas Carvalho VelosoEzquerra, TiberioRodrigues, Marco S.Ezugwu, SabastineRodrigues, RaquelFabián Gualdrón Reyes, AndrésRodríguez Mendoza, Ulises RuymánFábián, IstvánRodríguez Pascual, AlejandroFabre, BrunoRodriguez Torres, Claudia E.Fábrega, CristianRodriguez, Jose P.Fagadar-Cosma, EugeniaRodríguez-Lozano, Francisco JavierFagan, JeffreyRodygin, Konstantin S.Faísca Phillips, Ana MariaRogach, AndreyFakhrullin, RawilRogl, GerdaFalcó, AlbertoRogozhkin, S. V.Falk, Abram L.Roh, ChanghyunFalko, VladimirRoh, Kwang ChulFan, JiabingRoig, AnnaFan, JiepingRojas-Hernandez, Rocío EstefaníaFan, JiyangRojo, JoséFan, Lou-ZhenRokosz, KrzysztofFan, XuelingRoldán Carmona, CristinaFan, YiqunRoldán, Juan BautistaFan, YuanchengRoldán, PedroFancher, ChrisRolfe, Barbara E.Fanciulli, MarcoRoll, MarkFanelli, FiorenzaRoma, JoanaFang, BaizengRoman, AndresFang, JunRoman, MaciejFang, KegongRomar, HenrikFang, RunchenRombi, E.Fang, XiaoshengRomeira, BrunoFangueiro, JoanaRomero, AlbertoFanti, AlessandroRomero, Rosenberg J.Fantoni, AlessandroRondin, LoïcFaraldos, MarisolRoose, BartFaraudo, JordiRosa, LorenzoFarè, SilviaRosa, Luis GuerraFarhat, MohamedRosas, GerardoFarka, ZdeněkRosca, Daniel-IosifFarnesi, DanieleRosenzweig, Steven A.Fasoli, MauroRoshchupkin, Dmitry V.Fateixa, SaraRosi, MarzioFátima I.C., MiranteRos-Lis, JoseFatyeyeva, KaterynaRoslova, MariaFavier, LidiaRoso, MartinaFdez-Gubieda, Maria LuisaRossella, FrancescoFeás, XesúsRossetti, IleniaFebbraio, FerdinandoRossi, LorenzoFedorov, GeorgyRossi, MarcoFedorov, VladimirRossi, SilviaFeenstra, RandallRossin, AndreaFei, JinboRossner, PavelFejfar, AntonínRoszak, SzczepanFeldblyum, Jeremy I.Rotaru, AndreiFelgueiras, HelenaRotello, Vincent M.Feng, FudeRoth, Stephan V.Feng, JijunRothbauer, MarioFeng, JinkuiRoualdès, StéphanieFeng, Mei-LingRouco, VictorFeng, Sheng-WeiRoulia, MariaFeng, XiamingRoussey, MatthieuFeng, YanhuiRoy, AnupamFeng, YixuanRoy, SagarFeng, YongjunRoyo, MiriamFeng, YouzhiRozhina, ElviraFenwick, OliverRozhkova, NataliaFerdous, WahidRozynek, ZbigniewFernandes, António JoséRuaro, BarbaraFernandes, CarlosRubasinghege, GayanFernandes, FábioRubčić, MirtaFernandes, Maria HelenaRubini, SilviaFernandes, Susete NogueiraRubio Alonso, FaustoFernández Ramos, María DoloresRubio Alonso, JuanFernandez, CarlosRucandio, IsabelFernández, Juan PedroRuda, HarryFernández, PalomaRudawska, AnnaFernández, SusanaRudolf, R.Fernández-Arévalo, MercedesRuffato, GianlucaFernández-Gutiérrez, MarRuffino, FrancescoFernandez-Marchante, Carmen M.Rui, YukuiFerrando Soria, JesúsRuiz, SoniaFerrari, MaurizioRuiz-Hernandez, EduardoFerraro, AntonioRuiz-Rubio, LeireFerraro, GiaritaRumyantseva, MarinaFerreira, FilipeRunowski, MarcinFerreira, Paula M.Ruotolo, RobertaFerreiro-Vila, EliasRurack, KnutFerretti, ElenaRurali, RiccardoFiallo, MarinaRusen, EdinaFiandra, LuisaRusponi, StefanoFicek, MateuszRusso, Patrícia A.Fieber, Lynne A.Russo, TeresaFierascu, Radu ClaudiuRüther, ThomasFifere, AdrianRuths, MarinaFigueira, RitaRydosz, ArturFilarowski, AleksanderRykowska, IwonaFilice, MarcoRynkowski, JacekFilice, SimonaRyoo, Hyun-moFilip, JaroslavRyou, Jae-SukFilip, PeterRyu, Han-YoulFilipecki, JacekRyu, Ja-HyoungFilipescu, MihaelaRyu, JehwangFilippini, FrancescoRyu, Ju HeeFilippov, Andrey V.Ryu, SangwooFilippov, SergeyRyuzaki, SouFilonova, ElenaRzodkiewicz, WitoldFina, IgnasiSa, BaishengFini, PaolaSá, RosáliaFinšgar, MatjažSaad, Hosam A.Fiori, FabrizioSabantina, LiliaFiorica, CalogeroSabbatini, LuigiaFiorillo, AntoninoSaccoccio, MattiaFirdessa, RebumaSaccone, SalvatoreFisch, MichaelSachet, EdwardFischer, Inga AnitaSack, UlrichFischetti, Massimo V.Sadoh, TaizohFitta, MagdalenaSadovnikov, AlexandrFitter, JörgSadowska, BeataFlandin, LionelSadowska, KamilaFleury, EricSadowski, JanuszFlieger, JolantaSadrzadeh, MohtadaFlorea, AdrianSadykov, VladislavFlorea, IleanaSaeed, KhalidFlores, Ana I.Safaei, M. R.Florian Baron, CamiloSaha, BiswajitFlorian-Baron, CamiloSaha, Manik LalFlory, FrançoisSahoo, SurjitFobelets, KristelSahraoui, BouchtaFohlerová, ZdenkaSahu, Saura C.Fominski, V.Sahu, SushantFonseca, NelsonSaiki, ToshiharuFontana, MarcoSaikova, SvetlanaFontanesi, ClaudioSaini, NaurangFontecha-Cámara, María ÁngelesSajadifar, Seyed VahidFoo, SimonSajgalik, PavolForay, GenevieveSajkiewicz, PawełFormánek, PetrSakaguchi, HidetsuguFormela, KrzysztofSakaguchi, ToshikazuForrer, DanielSakai, JoeFortunati, IlariaSakellariou, GeorgeFoster, Kenneth R.Sakon, TakuoFoti, DoraSakthivel, Tamil SelvanFouda, AmrSalam, AkbarFountos, GeorgeSalaün, PascalFraga-García, PaulaSaleem, ImranFragoso, AlexSaleh, MazenFraleoni-Morgera, AlessandroSalim, MalindaFrances Monllor, JorgeŠalkus, TomasFrancesca, SelminSalmas, ConstantinosFrancesco Sgroi, MauroSalvati, EnricoFranco, RicardoSalzillo, TommasoFrangini, StefanoSamaras, IoannisFrank, IrmgardSambri, LetiziaFranke, DanielaSamiec, MarcinFrank-Kamenetskaya, Olga V.Samikannu, AjaikumarFranssila, SamiSammons, RLFranz, GerhardSan Andrés, María PazFratalocchi, AndreaSánchez Navarro, AmparoFraternali, FernandoSánchez, F.Fratoddi, IlariaSánchez-Chardi, AlejandroFraxedas, JordiSánchez-García, DavidFreakley, SimonSanctis, ShawnFreire, Juan J.Sandri, GiuseppinaFresch, BarbaraSandu, IonFreyria, Francesca StefaniaSang, YuanhuaFrielinghaus, HenrichSangiovanni, DavideFrka-Petesic, BrunoSanina, ViktoriyaFröhlich, EleonoreSanjuán, Miguel ÁngelFrömling, TillSankowski, DominikFrontera, CarlosSanna, SimoneFrontistis, ZachariasSansone, AnnaFruth, Victor OprisanSantamaria, AntxonFu, GengtaoSantamaría, Pedro AntonioFu, LiSantangelo, SaveriaFu, LiansheSanthanam, KSVFu, QiangŠantić, AnaFu, ZhuojiaSantoni, Marie-PierreFucikova, AnnaSantos, JoanaFuh, Lih-JyhSantos, PauloFujii, TakenoriSantos-Gómez, Lucía DosFujiki, MichiyaSantos-Ruiz, LeonorFujita, MorifumiSañudo, E. CarolinaFujiwara, AkihikoSanyal, DirthaFukata, NaokiSanz, RuyFukuda, KenjiroSapino, SimonaFukuda, MakotoSapkota, JanakFukumoto, Ken-ichiSaranin, Danila S.Fukushima, TakafumiSarantopoulou, EvangeliaFulgenzi, GianlucaSaravanakumar, KandasamyFun, LIM YeeSaremi, MehdiFurmaniak, SylwesterSarlin, EssiFusco, RobertaSarris, Ioannis E.Fusco, SalvatoreSarswat, Prashant K.Fuso, FrancescoSarto, FrancescaFutaba, Don NorimiSasaki, KenG. Kozlova, SvetlanaSasaki, SatoshiGábor, KatonaSasson, YoelGabrielson, Nathan P.Šatínský, DaliborGadipelli, SrinivasSato, HideyukiGaidukovs, SergejsSato, Shunsuke A.Gajovic, AndrejaSato, YukioGajski, GoranSatrapinskyy, LeonidGalamboš, MichalSatriano, CristinaGalateanu, BiancaSatyanaga, AlfrendoGaleotti, FrancescoSavara, Aditya AshiGalian, Raquel E.Savin, AlexanderGalinetto, PietroSaviot, LucienGallego, AntolinoSawai, JunGallei, MarkusSawicki, MaciejGallo, JuanScaffaro, RobertoGallo, MonicaScaini, DenisGallo, SalvatoreScalese, SilviaGalstyan, VardanScalise, EmilioGamal El-Din, Tamer M.Scandurra, GraziellaGambardella, AlessandroScarano, AntonioGamulin, OzrenScarano, DomenicaGan, Chee KwanScardaci, VittorioGan, LihuaScardi, PaoloGan, Yong X.Scarselli, ManuelaGan, ZhixingScarso, AlessandroGandolfi, MarcoSchaefer, Hans-EckhardtGane, PatrickScheibe, BłażejGanguly, SayanScheiner, SteveGanikhanov, FeruzSchellenberg, PeterGao, BoScheper, ThomasGao, Hongyu (Larry)Schepler, KennethGao, JinSchiavi, Pier GiorgioGao, JingjingSchick, ChristophGao, YachenSchiek, ManuelaGao, YananSchierbaum, Klaus DieterGao, YaoSchilardi, PatriciaGao, YongliSchindler, IvoGaponik, NikolaiSchiraldi, David A.Garaio, EnekoSchirhagl, RomanaGarbovskiy, YuriySchlosser, StefanGarcia Amorós, JaumeSchmedake, Thomas A.García Díaz, IreneSchmelzer, Jürn W.P.García Mandayo, GemmaSchmidt, BernhardGarcía Rodríguez, JuanSchmidt, RainerGarcía Ruiz, Francisco JavierSchmidt, Wolf-geroGarcía Suárez, Víctor ManuelSchnabel, ManuelGarcia, Andres SanzSchneckenburger, HerbertGarcía, José MiguelSchneider, JennyGarcia, Maria Isabel DiezSchneider, NathanaelleGarcía, Miguel TorresSchneider, RaphaëlGarcía-Antón, JordiSchöenhals, AndreasGarcia-Bernabé, AbelScholz, FerdinandGarcia-Deibe, AnaSchönecker, AndreasGarcia-Garcia, Francisco J.Schröder, SvenGarcia-Granada, Andrés AmadorSchröder, ThiesGarcia-Granda, SantiagoSchroeder, GrzegorzGarcía-López, Elisa I.Schroeder, Marshall A.García-Loureiro, AntonioSchüler, MichaelGarcía-March, Miguel ÁngelSchulz, AlexanderGarcía-Miquel, HectorSchulz, BernhardGarcía-Pérez, Blanca EstelaSchulz, FlorianGarcia-Pomar, Juan LuisSchulz, MarkGarcia-Torres, JoséSchulz, WolfgangGardikis, KonstantinosSchusser, Gerald FritzGardiner, PhilipSchütt, FabianGareev, KamilSchwaminger, SebastianGargiulo, ValentinaSchwarz, KarlheinzGarmire, DavidŚcibisz, IwonaGarnweitner, GeorgScognamiglio, VivianaGaroli, DenisScomparin, AnnaGarriga, RosaScotognella, FrancescoGarrigue, PhilippeScozzafava, AndreaGarroni, SebastianoScrimin, PaoloGas, PiotrScuderi, VivianaGatica, José M.Sebastian, VictorGatti, TeresaSecenji, AleksandarGatto, EmanuelaSecu, Elisabeta-CorinaGauden, PiotrSecu, MihailGaudry, ÉmilieSecuianu, CatincaGaumet, Jean-JacquesSedlačík, MichalGautam, BhojSedmidubský, DavidGautam, SiddharthSeefeldt, MarcGavini, ElisabettaSeh, Zhi WeiGavrilova, NataliaSeibt, MichaelGazda, MariaSeida, YoshimiGębara, PiotrSeisenbaeva, GulaimGebicki, JacekSeitz, W. RudolfGębicki, JacekSelimefendigil, FatihGéczy, AttilaSelishchev, DmitryGedvilas, MindaugasSelke, MatthiasGeelhaar, LutzSelvasembian, RangabhashiyamGeints, Yurii ESelyanchyn, RomanGeissler, DanielSementa, LucaGelardi, Franco MarioSemenyuk, PavelGelbstein, YanivSemih, EnerGellini, CristinaSemisalova, Anna S.Geng, DechaoSempionatto, Juliane R.Geng, Hong ZhangSenetakis, KonstantinosGenorio, BostjanSenge, MathiasGentile, PaolaSeo, Dae-ShikGentili, DenisSeo, DaisukeGeorgakilas, AlexandrosSeo, Sang-WooGeorgantzinos, Stelios K.Seo, Young-KwonGeorge, Thomas F.Seong, GimyeongGeorgiev, DankoSepúlveda, BorjaGeorgieva, RadostinaSeradjeh, BabakGeorgii, RobertSeravalli, LucaGeorgopanos, ProkopiosSeres, JozsefGeraldes, CarlosSerfa Juan, Ronnie O.Gercsi, ZsoltSergeev, AlexanderGerislioglu, BurakSergeev, MaximGeso, MoshiSerna-Galvis, Efraím A.Gesuele, FeliceSerpooshan, VahidGeszke-Moritz, MałgorzataSerrano, AidaGettemans, Jan GettemansSeshadri, Ramachandran ChidambaramGhalambaz, MohammadSetzer, William N. Ghalkhani, MasoumehSevilla-Morán, BeatrizGhanekar, AlokSevostyanov, MikhailGhanotakis, Demetrios FSgarzi, MassimoGharibshahi, ElhamSha, JianjunGheju, MariusShabatina, TatyanaGhenuche, PetruShaginyan, V. R.Gheorghiu, EugenShah, Nilam CGhezzi, FrancescoShahiduzzaman, M.Ghica, CorneliuShaik, Mohammed RafiGhica, Mariana EmiliaShainsky, JannaGhidelli, MatteoShakeel, FaiyazGhinassi, BarbaraShakhaoath, Md.Ghione, GiovanniShakya, AkhileshGhodake, GajananShalan, Ahmed EsmailGholami, PeymanShalev, GilGhomi, MahmoudShamonin, MikhailGhosh, DebanjanaShan, WenpoGhosh, SantoshShankar Rao, Doddamane Sreenivasamurthy ShankarGhosh, SubrataShankar, KarthikGiancane, GabrieleShao, GuoshengGianferrara, TeresaShao, HaoGiannazzo, FilippoShao, TaoGiannopoulos, Georgios I.Shao, WeiGiannossa, Lorena CarlaShapter, JoeGibot, M. PierreSharkeev, YuriGibson, ChristopherSharma, Dinesh KumarGidwani, KamleshSharma, PriyankaGigante, Giovanni E.Sharma, YogeshGil Bravo, AntonioShaula, AliaksandrGil-Escrig, LidónShavandi, AminGindre, DenisShcharbin, DzmitryGinestra, PaolaShearer, CameronGinzburg, PavelShehata, NaderGioffré, MarianoSheka, Elena F.Gionco, ChiaraShelushinina, Nina G.Giorgetti, LuciaShemelya, CoreyGioti, MariaShen, Hui-ShenGiovannetti, RitaShen, JianleiGiovannini, GiorgiaShen, JianliangGirard, Hugues A.Shen, XiGirtan, MihaelaShen, XiaopingGîrțu, Mihai A.Shen, YanbaiGiubileo, FilippoShende, ChetanGiunchedi, PaoloShenderovich, Ilya G.Giunta, GaetanoSheng, HuaxinGizdavic-Nikolaidis, MarijaSheng, RuilongGjokas, MargaritisShestopalov, Michael A.Gjörloff-Wingren, AnetteShi, BojingGladyszewski, GrzegorzShi, DiGliemann, HartmutShi, FengGlomm, Wilhelm RShi, Jin-XingGloria, AntonioShi, QiongGłuchowski, PawełShi, RuiGlukhova, Olga E.Shi, Shih-ChenGlüsen, AndreasShi, Xiao-LeiGlushko, OleksandrShi, YongqianGnade, Bruce E.Shi, ZhifengGnyba, MarcinShibaev, PetrGodinho, Maria HelenaShichalin, OlegGogos, AlexanderShim, GayongGokarna, Anisha S.Shim, Jun HoGolding, JonShimanouchi, ToshinoriGoldoni, MatteoShimizu, TaroGoldthorpe, Irene A.Shimoi, NorihiroGolewski, Grzegorz LudwikShin, Bo-SungGolewski, PrzemysławShin, Chae-HoGolgovici, FlorentinaShin, Jun-HwanGolobič, IztokShin, SunmiGolub, IgorShin, Weon HoGolubnitschaja, OlgaShinde, Surendra KrushnaGomes, Henrique LeonelShinji, KawabataGomes-da-Silva, Lígia C.Shinoka, ToshiharuGómez Cámer, Juan LuisShinozaki, KenjiGomez Cerezo, Maria NatividadShiomori, KoichiroGomez, JulioShirahata, NaotoGómez-Graña, SergioShirai, HajimeGomez-Lazaro, MariaShirai, KouunGomila, GabrielShiroma, Wayne A.Gomonay, OlenaShironita, SayokoGonçalves, Helena M. R.Shirvani Moghaddam, KamyarGonçalves, RenatoShishkin, IvanGondek, LukaszShivakumar, Vibhav BharadwajGondolini, AngelaShiver, LembitGong, HaiboShklover, JenyGong, PeiweiShlyakhtin, Oleg A.Gontrani, LorenzoShneck, Roni Z.Gonzales, Ralph RollyShoji, SatoruGonzalez, Juan DiazShoujun, ZhuGonzalez-Gutierrez, JoaminShoute, Lian C. T.Gonzalez-Leal, J. M.Shtepliuk, IvanGonzález-Legarreta, LorenaShukla, SourabhGopalan, SaianandShukla, Sudheesh K.Gordeev, SergeyShulga, YuriyGordon, Wesley O.Shurpik, DmitriyGorin, DmitryShvartsman, VladimirGorji, Nima E.Sibatov, Renat T.Gorodkiewicz, EwaSibirev, NickolayGorte, Raymond J.Sicard-Roselli, CécileGorzelanny, ChristianSiddiqui, MasoomGoseki, RaitaSideratou, ZiliGossuin, YvesSiegel, JakubGoswami, MukundaSiegel, JanGoswami, NirmalSierra, Josep M.Göthelid, MatsSievers, SibylleGotić, MarijanŠiffalović, PeterGoto, YosukeSigalotti, L. Di G.Gottwald, AlexanderSigle, WilfriedGoula, Maria A.Signore, GiovanniGovan, JosephSignorini, RaffaellaGovindan, BharathSilkin, VyacheslavGovoni, MarcoSilva, BrunoGözde, TütüncüoǧluSilva, FranciscoGracia Lostao, Ana IsabelSilva, Maria JoaoGracin, DavorSilva, Maria ManuelaGradov, Oleg M.Silva, MónicaGránásy, LászlóSilva, SóniaGranroth, SariSilveira, CéliaGrassani, DavideSim, UkGrasset, FabienSima, FelixGrassi, AlfonsoSimanjuntak, Firman MangasaGrassi, MarioSimeonidis, KonstantinosGrasso, SalvatoreSimeonov, VasilGravel, EdmondSimion, CristianGraves, JosephSimion, Cristian EugenGrazioli, CesareSimka, WojciechGreabu, MariaSimon, JohnGreco, FrancescaSimoncelli, SabrinaGric, TatjanaSimončič, BarbaraGrigalevicius, SauliusSimonpietro, AgnelloGrigoriev, FedorSimova-Stoilova, Lyudmila PetrovaGrijalvo, SantiagoSimserides, ConstantinosGrishina, MariaSin, Jin-ChungGristina, RobertoSinebryukhov, Sergey LeonidovichGrivel, Jean-ClaudeSing, Swee LeongGromov, Alexander AlexandrovichSingh, Ajay VikramGrosjean, ThierrySingh, Ajaya KumarGross, Andrew JamesSingh, DavidGrosso, ClaraSingh, MahiGrueso, EliaSingh, NeenuGrugeon, SylvieSingh, PardeepGrumezescu, Alexandru MihaiSingh, Rajan KumarGrumezescu, ValentinaSingh, RanbirGruzdev, MatveySingh, SubhashGrybos, JoannaSinha, Sudarson SekharGrym, JanSinitsyna, Olga A.Grywalska, EwelinaSinn, GerhardGrzyb, JoannaSioutopoulos, DimitriosGrzyb, TomaszSipos, PálGu, JunweiSipponen, MikaGu, XuehongŠírová, MiladaGu, ZhanjunSiska, FilipGu, Zhi-GangSivanandam, SivasankaranGuan, JingqiSivaramapanicker, SreejithGuascito, Maria RacheleSkapin, Sreco D.Gubala, VladimirSkenderovic, HrvojeGuda, Alexander A.Skirtach, AndreGudkov, SergeySkoko, ŽeljkoGudmundsson, Jon TomasSkonieczna, MagdalenaGudmundsson, VidarSkorik, Yury A.Güell, FrankSkotak, MaciejGueorguiev, Gueorgui KostovSlavič, JankoGuerin, KatiaSlavov, AntonGuerrero-Pérez, M. OlgaSloan, JeremyGui, YingangSlodek, AnetaGuidoin, RobertSmarandache, AdrianaGuidotti, MatteoSmith, Thomas J.Guilemany, Josep MariaSmoliner, JuergenGuina, MirceaSmulko, JanuszGuiot, CaterinaSnytnikov, Pavel ValerievichGulati, KaranSoares, SofiaGulbinas, VidmantasŠob, MojmírGunawan, PoernomoSobola, DinaraGundlach, LarsSobolev, ArkadijGun’ko, Vladimir M.Socaci, CrinaGuo, BingSocol, MarcelaGuo, HuSocoliuc, VladGuo, JingSoengas, RaquelGuo, JinxinSogorb Sánchez, Miguel ÁngelGuo, PingSohn, YoungkuGuo, QiuquanSoin, NavneetGuo, ShishangSokolnicki, JerzyGuo, WeihongSokolov, IgorGuo, XingzhongSolin, NiclasGuo, YanchuanSolis, Rafael R.Guo, YaoSolodovnik, MaximGuo, YongfuSołoducho, JadwigaGuo, Zhan-HuSologubenko, AllaGuo, ZhinanSolovieva, AnastasiyaGupta, SanjuSolyanikova, Inna P.Gupta, SiddharthSome, SurajitGupta, TusharSon, Jong YeogGurevich, EvgenySon, Seung UkGurevich, LeonidSone, HidekoGurioli, MassimoSong, HaoGurung, AshimSong, HyungjunGuselnikova, Olga AndreevnaSong, JingfengGusev, AlexanderSong, JizhongGuskova, OlgaSong, JunGusmão, RuiSong, Ping’anGustafsson, AndersSong, ShipingGustav, BihlmayerSong, SunghoGutierrez, Mar FernandezSong, YingzeGutzov, StoyanSong, Young MinGuzek, DominikaSong, YuGuzmán, EduardoSong, YujunGuzman, MarceloSoo Min, HwangGuzman-Puyol, SusanaSoon, Jong JeongGyorgy, EnikoSoos, Zoltan G.Gyurcsik, BelaSopha, HannaGzara, LassaadSorli, BriceHa, Chang-SikSorrentino, LuigiHa, Don-HyungSosnowski, MarcinHa, Ji WonSotiropoulos, SotirisHackenberg, StephanSoto Rodriguez, Paul Eduardo DavidHadavinia, HomayounSoto, FernandoHaddadi, AbbasSoto, ManuHaddadi, Seyyed ArashSoto, PaulHaddour, NaoufelSoudagar, Manzoore Elahi M.Hadermann, JokeSour, AngéliqueHadjadj, AomarSousa, FláviaHadjikakou, Sotiris KSouto, ElianaHadziioannou, GeorgesSoveral, GraçaHahn, Jae RyangSovinco, FabioHai, Pham NamSpagnuolo, GianricoHajra, SugatoSpalek, JozefHalet, Jean-FrançoisSpanò, CarmelinaHall, SteveSpanopoulos, IoannisHallam, TobySparnacci, KatiaHalonen, NiinaSpasic, DraganaHama, SusumuSpătaru, NiculaeHamad-Schifferli, KimberleySpencer, BenHamam, YskandarSperanza, GiorgioHamankiewicz, BartoszSpinelli, GiovanniHamdan, AhmadSpinsante, SusannaHampp, Norbert A.Spiridigliozzi, LucaHan, BaoguoSpirito, DavideHan, ChaoSpirk, StefanHan, Dong-PyoSpiros, AnastasiadisHan, HeyouSpivak, YuliaHan, Il KiSpizzo, FedericoHan, Jae-HeeSplinter, RobertHan, JingquanSportelli, Maria ChiaraHan, Joong TarkSprenger, KaylaHan, Jun HyunSprenger, StephanHan, PingpingSprynskyy, MyroslavHan, WenboSramkova, MonikaHanaor, Dorian A.H.Sreekanth, K.V.Handke, BartoszSrinivasa Rao, SunkaraHanifehpour, YounesSrivastava, G.P.Haniu, HisaoSrivastava, VarshaHanks, TimothySrivastava, Yogesh KumarHansen, WolfgangStadnichenko, AndreyHantanasirisakul, KanitStafeev, SergeyHao, NanjingStafford, James L.Hao, QingStagi, LuigiHaponiuk, Józef T.Stahl, FrankHaque, ArifulStaicu, AngelaHaraguchi, NaokiStamate, EugenHarja, MariaStamatin, IoanHärk, EneliStamplecoskie, KevinHarmer-Bassell, SarahStangel, ChristinaHarničárová, MartaStanisz, BeataHarrell, William R.Starikov, SergeiHartman, Matthew C.T.Starsich, FabianHasal, PavelStarzyński, Rafał R.Hasan, Md NazmulStathi, PanagiotaHasani, AmirhosseinStefan, MariusHasegawa, GeorgeStefan, RazvanHasegawa, MakotoStefan-Andrei, IrimiciucHassabo, AhmedStefanelli, ManuelaHassanpour, AmirŠtefanić, Petra PeharecHassanzadeh-Aghdam, Mohammad KazemSteinberg, IvanaHatayama, ShogoSteinhauer, StephanHattori, Azusa N.Stellas, DimitriosHattori, Haroldo T.Stelmachowski, PawełHatzikraniotis, EvripidisStelzer, FranzHaupt, MichaelStenzel, OlafHawash, ZaferStepanenko, Olesya V.Hawrylak, PawelStępnik, MaciejHay, SamStępniowski, WojciechHayashi, HisashiStergar, JanjaHayashi, NorikoStern, RaivoHayashi, TakuyaSterzyński, TomaszHayrapetyan, D.B.Stetsyshyn, YurijHazra, DibyenduSthal, FabriceHe, ChuanglongStine, Keith J.He, DapingStipa, PierluigiHe, GuanjieStirner, ThomasHe, JianzhouStobiecka, MagdalenaHe, JiaruiStöckelhuber, Klaus WernerHe, LiangcanStodolak, EwaHe, MaoshuaiStöger-Pollach, MichaelHe, NafeiStoikov, Ivan IvanovichHe, QiyuanStojek, ZbigniewHe, YongStoklas, RomanHe, YurongStolarczyk, AgnieszkaHe, ZhubingStoleru, ElenaHector, MarkStolojan, VladHedrich, JanaStolov, Andrei A.Hegedűs, CsabaStomeo, TizianaHegedűs, LászlóStopic, SreckoHegmann, EldaStorsberg, JoachimHeiderhoff, RalfStrakosas, XenofonHeier, JakobStrambini, LucanosHeikinheimo, MattiStrankowski, MichalHeim, FredericStratakis, ManolisHeimann, RobertStrbak, OliverHeinrich, BenoîtStrečka, JozefHeitmann, TomStreimikiene, DaliaHelseth, LarsStrelniker, YakovHenderson, ChristopherStrout, Douglas L.Henkel, CarstenStruga, MartaHeo, JinseokStrukul, GiorgioHeo, SoowonStrunskus, ThomasHergenröder, RolandStrzałkowski, KarolHeris, Saeed ZeinaliStrzemiecka, BeataHermida, Jesús BarrioStylianakis, Minas M.Hernández Creus, AlbertoSu, Chia-HaoHernández López, LeonorSu, FengmeiHernando, A.Su, QingHerrera-Melián, José AlbertoSu, Zheng-YuanHeuer-Jungemann, AmelieSuarez, TeresaHeuken, MichaelSuber, LorenzaHeyn, ChristianSubhan, FazleHijikata, YasutoSubramanian, PalaniappanHill, JonathanSuckeveriene, RanHilleringmann, UlrichSuffczyński, JanHimly, MartinSugawara-Narutaki, AyaeHincapie, Rafael E.Suh, Jin-YooHinds, GarethSuh, Jun MinHinode, HirofumiSujecki, SlawomirHiorns, RogerSulaiman, Ghassan M.Hippler, RainerSulka, Grzegorz DariuszHirata, TomotakeSullivan, James A.Hirosawa, ShoichiSumboja, AfriyantiHirscher, MichaelSummers, HuwHirtz, MichaelSun, BaiHisada, KenjiSun, Chia-LiangHíveš, JánSun, DeyanHix, GarySun, HanjunHng, Huey HoonSun, JihongHo, JamesSun, JinhuaHo, Wen-JengSun, JunweiHo, Yen‐PengSun, Kien-WenHobbie, Erik K.Sun, LeiHobbs, RichardSun, LinfengHodoroaba, Vasile-DanSun, LuzhaoHoffmann-Eifert, SusanneSun, MengHofmeister, AnneSun, MengtaoHogan, ConorSun, QiHogan, James D.Sun, QianHokamoto, KazuyukiSun, Shih-JyeHollmann, AxelSun, TaoHolmes, ChristopherSun, WenHolst, BodilSun, WenhongHolz, Richard C.Sun, XupingHoma, JoannaSun, YifeiHomaeigohar, ShahinSun, YilinHoms, NarcisSun, ZhengmingHong, Chih ChiangSundarrajan, SubramanianHong, ChunyanSundqvist, BertilHong, HaipingSuñol, Joan-JosepHong, MinghuiSuo, GuoquanHong, SukjoonSurdo, SalvatoreHong, Woong-KiSurendran, SubramaniHoon, Stephen R.Surmenev, RomanHorbett, Thomas ASuryanarayana, PhanishHortigüela, María J.Susarrey-Arce, ArturoHorvat, JosipSuturin, SergeyHou, JungangSuzudo, TomoakiHourdakis, EmmanouelSuzuki, NorihiroHouska, JiriSuzuki, YoshioHouston, Zachary H.Sverdlov, ViktorHowell, BobSvirko, YuriHowes, PhilipSvirshchevskaya, Elena V.Hrabovsky, MilanŠvrček, VladimirHristoforou, EvangelosŚwierczek, JanHrnjak-Murgić, ZlataŚwietlik, RomanHsiao, Chung-DerSydänheimo, LauriHsiao, Fu LiSyrgiannis, ZoisHsiao, VincentSyrrokostas, GeorgeHsiao, Yu-JenSysoev, VictorHsieh, Chih-ChunSzabó, GyörgyHsieh, Ya-PingSzabó, TamásHsu, Cheng-CheSzakmany, GergoHsu, Cheng-HsunSzala, MateuszHsu, Chih-pingSzałowski, KarolHsu, Hung-PinSzczepanowicz, KrzysztofHsu, Jin-ChenSzczurek, AndrzejHsu, Jin-CherngSzekely, GyorgyHsu, Julia W. P.Szewczyk, RomanHsu, Yu-KueiSzłyk, EdwardHsueh, Han-YuSzoszkiewicz, RobertHsueh, Yi-HuangSzperlich, PiotrHu, AnmingSzulczewski, GregoryHu, FangzhongSzustakiewicz, KonradHu, HanSzybowicz, MiroslawHu, JiansheSzyja, Bartłomiej M.Hu, Jin-JiaSzymborski, TomaszHu, JinmingSzymczyk, AnnaHu, KuanTabacaru, AurelHu, MingTabakova, TatyanaHu, RunTăbăran, Flaviu AlexandruHu, WenbinTabernero, VanessaHu, XiaobinTabish, Tanveer AhmadHu, YangTaboryski, RafaelHuang, ChangshuiTaccola, SilviaHuang, Chao-MingTadyszak, KrzysztofHuang, Chih-ChingTae-Jun, HaHuang, Chih-FengTaghinejad, HosseinHuang, Chun-YingTagliaferro, AlbertoHuang, Chun-YuanTaglietti, AngeloHuang, DaliTaguet, AurélieHuang, E-WenTahara, HironobuHuang, Genin GaryTaheri, ShimaHuang, Hung JiTaheri-Qazvini, NaderHuang, JiaruiTait, NiallHuang, JinTak, YongsugHuang, JingfengTakahashi, KaitoHuang, JunjianTakahashi, KenjiHuang, Keng-ShiangTakahiro, KatsumiHuang, Kuo-ChengTakano, ToshiyukiHuang, QijinTakashi, YanagisawaHuang, QiliangTakashima, ToshihiroHuang, RuomengTakeda, YasunoriHuang, ShengTakeguchi, TatsuyaHuang, ShengxiTakei, HiroyukiHuang, ShuiquanTakeuchi, TomonariHuang, TaizhongTakoudis, Christos G.Huang, XiaoyuTaleb, AbdelhafedHuang, YongTalebian, SepehrHuang, YongchaoTaler, JanHuang, Zheng-HongTalneau, AnneHuang, ZhiyuanTalroze, Raisa V.Hubbe, Martin A.Țălu, ȘtefanHubička, ZdeněkTamaki, RyoHuc, VincentTamargo, MariaHueso, Jose L.Tamulevičienė, AstaHueso, Jose MarquésTamulevicius, SigitasHughes, Robert A.Tamura, RuiHühne, RubenTamus, Zoltán ÁdámHuignard, Jean-PierreTan, Chee-KeongHumbert, ChristopheTan, JunjunHuminic, AngelTan, Wai KianHuminic, GabrielaTanabe, KatsuakiHung, Tai-FengTanaka, GengoHunge, Yuvaraj M.Tanaka, MasatoHunter, Wayne BrianTandecka, KatarzynaHuntosova, VeronikaTang, LihongHussain Ibupoto, ZafarTang, PengyiHussain, AfzalTang, WeiHussain, ShahidTangl, StefanHussainova, IrinaTaniguchi, JunHuynh, VienTao, WeiHwang, Dae-seokTao, YunwenHwang, Seong-JuTaradaj, JakubHwang, SooyeonTarasov, AlexeyHwang, Taek YongTarasov, IvanHwang, YuhoonTarasov, MichaelHyung-Seok, KimTarasova, Nataliia A.Iaccino, EnricoTardy, BlaiseIacob, CiprianTariq, MohammadIacopi, FrancescaTarnowski, KarolIafisco, MicheleTarrés, QuimIanchis, RalucaTashima, DaisukeIanieri, AdrianaTasiopoulos, AnastasiosIannace, GinoTasis, DimitriosIannazzo, DanielaTassinari, FrancescoIannuccelli, ValentinaTassinari, RobertaIasiello, MarcelloTateno, HiroyukiIatì, Maria AntoniaTatlock, GordonIatridi, ZacharoulaTaub, AlanIatrou, HermisTaurino, AntoniettaIatsunskyi, IgorTavares, AnthonyIchikawa, MasakazuTavares, Carlos Jose MacedoIchikawa, YoTavkhelidze, AvtoIconaru, Simona LilianaTay, Chor YongIdrees, FaryalTay, Zhi WeiIezzi, GiovannaTayabali, AzamIglesias, EmiliaTealdi, CristinaIglesias, MònicaTebyetekerwa, MikeIglesias-Martínez, EmiliaTeghil, RobertoIgnat, MariaTeixeira, BárbaraIguchi, FumitadaTeixeira, João PauloIkeda, HiroshiTeixeira, Paulo I. C.Ikegami, MasashiTeixeira, RóbsonIl, JeonTeleki, AlexandraIlahi, BouraouiTempleton, Joseph L.Ilaš, JanezTeng, Qian YongIlg, PatrickTeng, Tun-PingIlia, GheorgheTeodorescu, Valentin ŞerbanIlias, MiroslavTeodoriu, CatalinIllarionov, Yury YuryevichTercjak, AgnieszkaIllés, BalázsTermentzidis, KonstantinosIllia, DobrydenTero, RyugoIllias, Hazlee Azil BinTerrasson, VincentIlnytskyi, JaroslavTertis, MihaelaIl’yushin, Mikhail A.Tertiş, MihaelaImamura, RyuTessore, FrancescaImperatore, RobertaTestarelli, LucaImprota, GiovanniTetean, RomulusIndra, ArindamTeterycz, HelenaInoue, ShuheiTeyssier, JeremieInsausti, MaiteThakur, AnukulInsepov, Zinetula (Zeke)Thakur, GarimaIoan Voicu, StefanThakur, Vijay KumarIoannides, TheophilosThang, Tran QuyetIoele, GiuseppinaThangavel, ChellappagounderIoelovich, Michael JacobThangavel, RanjithIon, Rodica-MarianaThavasi, VelmuruganIonita, GabrielaTheodorakis, PanagiotisIonita, PetreTheodorou, ChristoforosIorio, Carlo SaverioThill, AntoineIqbal Faruque, Mohammad RashedThomas, Selvin P.Iqbal, TahirThomas, SibyIrrera, AlessiaThompson, MichaelIsaad, JalalThompson, Richard L.Isac, LuminitaThomsen, KnudIsaiev, Mykola V.Thongbai, PrasitIshida, TamaoThoury, MathieuIslam, Md RashadulThulstrup, Peter W.Island, J. O.Thunemann, MartinIsola, GaetanoTian, FurongIsono, TakuyaTian, HaoIsozaki, KatsuhiroTian, JiangweiIto, HiroakiTibbetts, Katharine MooreItoh, TamitakeTiberto, PaolaIuliana, StoicaTien, Chuen-LinIvankov, Dmitry N.Tien, Li-ChiaIvanov, AlexeyTiggelaar, Roald M.Ivanov, DmitryTigran, VartanyanIvanov, Ilia N.Timoshenko, ViktorIvanov, Vladimir K.Tirozzi, BrunelloIvanov, Yuri D.Tišler, ZdeněkIvanova, BojidarkaTiwari, Arjun PrasadIvanova, ElenaTjong, Sie ChinIvanova, NataliaTkac, JanIvars-Barceló, FranciscoTkáč, JánIvchenko, Pavel V.Tkach, OleksandrIverson, Nicole M.Toby, BrianIwański, MarekTodorov, RossenIzu, NoriyaTofan, LaviniaJabbarzadeh, AhmadToffano, ZenoJabłońska, KrystynaTojo, ConchaJacak, JaroslawTokunaga, EijiJacak, LucjanTokura, YasuhiroJachowicz, RyszardTomasiunas, RolandasJackson, Mark JamesTomašovičová, NatáliaJackson, NathanTombacz, EtelkaJacquemin, DenisTomčík, PeterJacquemin, JohanTomm, JensJadczak, Joanna N.Tommasi, ImmacolataJafarinejad, ShahryarTomokiyo, YoshitsuguJagiełło, JoannaTompos, AndrásJagminas, ArunasTomšič, BrigitaJakła, SlawomirToncheva, AntoniyaJakubec, PetrToney, James E.Jakubik, Wiesław P.Tong, LianmingJamting, AsaTong, WangshuJan Laskowski, ŁukaszTong, YexiangJana, AtanuTopinka, JanJanas, DawidTopoglidis, EmmanuelJanda, PavelTorbeev, VladimirJang, Ho WonTorcates, Lourdes RivasJang, HongjeTorigoe, KanjiroJang, JaewonTornabene, FrancescoJang, Ji-sooToro, Roberta GraziaJankowski, Nicholas RobertToropov, AlexeyJanowska, IzabelaTorres Marques, AntonioJanowski, MiroslawTorres Vaamonde, José EnriqueJanssens, EwaldTorroba, TomasJanus, WladyslawTortello, MauroJanuszkiewicz-Lewandowska, DanutaTosello, GuidoJanuszko, AdamTosoni, SergioJapip, SusiloTotu, EugeniaJarząbek, BożenaTownley, HelenJašek, OndřejToyir, JamilJasiński, MariuszTozzini, ValentinaJasiński, RadomirTräbert, ElmarJastrzębska, Agnieszka MariaTrallero-Giner, CarlosJavadian, HamedrezaTrammell, Scott A.Javaid, RahatTran, FabienJaworska, MariaTravlos, IliasJedrkiewicz, OttaviaTremblay, Jean ChristopheJędrysiak, JarosławTremlová, BohuslavaJędrzejczyk, Roman J.Trens, PhilippeJeerapan, ItthiponTretiakov, Konstantin V.Jelliss, PaulTriantafyllidis, KonstantinosJen, Yi-JunTrindle, CarlJena, Himanshu SekharTrinh, ThuatJeng, Ming-JerTrioni, Mario ItaloJenkins, DavidTripathi, ManojJenkins, Samir V.Trivedi, RahulJeon, Hwan-JinTrohidou, KalliopiJeon, JonghoTrubiani, OrianaJeon, SangminTrukhanov, Alex V.Jeong, HyominTrukhanova, Ekaterina L.Jeong, MunseokTruong, Nghia P.Jerzykiewicz, LucjanTruong, Vi-KhanhJezierska, AnetaTrushechkin, AntonJezierski, JanTrusov, PeterJha, Prafulla K.Truszkiewicz, ElżbietaJha, RajveerTrybuła, ZbigniewJi, Hai-Feng (Frank)Tryk, DonaldJi, VincentTsai, Chen-YenJi, WeiTsai, Dung-shengJia, GuangTsai, Ming-JunJia, GuohuaTsamis, ChristosJia, MingjunTsay, CHien-YieJia, QuanliTselev, AlexanderJia, XudongTseng, Hui-hsinJian, WurongTseng, Wei-TauJiang, Bang-PingTsiafoulis, Constantinos G.Jiang, KailiTsikritzis, DimitrisJiang, LeyongTsimpanogiannis, Ioannis N.Jiang, ShanTsivintzelis, IoannisJiang, TaoTsoukalas, DimitrisJiang, XuchuanTsouknidas, AlexanderJiang, XueliangTsubaki, ShuntaroJiang, ZaixingTsud, NataliyaJiang, ZhangTsuji, YasuhideJiang, ZiwenTsujiguchi, TakuyaJiao, ShujieTsukerblat, BorisJicsinszky, LászlóTsutsui, MakusuJiménez Blanco, José L.Tsutsumi, OsamuJiménez, JuanTsvetkov, NikolaiJiménez-Molinos, FranciscoTsyganenko, Alexey A.Jiménez-Solano, AlbertoTu, RongJin, LeiTu, YifengJin, QinghuiTuccitto, NunzioJin, RenxiTuci, GiuliaJing, GuangyinTudor Matea, CristianJing, WenwenTumkin, Ilya I.Jishkariani, DavitTung, Shih-HuangJmerik, ValentinTung, Tran ThanhJo, ChangshinTurco, AntonioJo, JanggunTürk, MichaelJo, Sae ByeokTurkmen, MustafaJo, Young MinTurner, Paul E.João Pires, Da SilvaTurner, Raymond J.Johánek, ViktorTuros, EdwardJohannes, Hans-HermannTuru, GáborJohansson, ChristerTveryanovich, AndreyJohari, KhairiraihannaTyliszczak, BożenaJohlin, EricTyschenko, Ida E.John, ŁukaszTzeng, Tzuen-RongJohnson, Noah RayTzeng, YonhuaJohnsson, MatsTzetzis, DimitriosJohnston, Linda J.Tziveleka, Leto-AikateriniJoo, YonghoTzounis, LazarosJordán, ManuelUchaikin, SergeyJordao, LuisaUchida, SatoshiJoshi, NiravUddi, MruthunjayaJoshi-Imre, AlexandraUddin, Mohammad AfsarJouault, B.Uen, Wu-YihJoubert, OlivierUgo, PaoloJouini, NoureddineUhrig, Götz S.Jovanović, SvetlanaUjihara, MasakiJovanovski, VaskoUllrich, CarstenJózefczak, ArkadiuszUm, Doo-SeungJóźwiak, TomaszUngureanu, CameliaJuang, Jia-YangUnnithan, Ranjith RajasekharanJue, Melinda L.Unterreiner, Andreas-NeilJulian-Lopez, BeatrizUrase, TaroJulien, Christian M.Urban, FrancescaJulkapli, Nurhidayatullaili MuhdUrbanczyk-Lipkowska, ZofiaJun, Young-SiUrsu, CristianJung, Byung MunUrsu, LauraJung, DaesooUshakov, NikolaiJung, Gang SeobUsui, HidetomoJung, Jae HwanUsui, HiroyukiJung, JaehanUvarov, NikolaiJung, Ji-WonUznanski, PawelJung, Julie Cathy Antoinette OdileVacca, AnnalisaJung, OleVaculovicova, MarketaJung, YongwonVadahanambi, SridharJungjohann, Katherine L.Vafaei, SaeidJuodkazis, SauliusVaghjiani, Ghanshyam L.Juodkazytė, JurgaVaiano, VincenzoJurado, Amalia S.Vakros, JohnJurasekova, ZuzanaValagiannopoulos, ConstantinosJurca, TitelValais, Ioannis G.Jurj, Ancuţa MariaValášková, MartaJurkin, TanjaValente, JoãoJursich, GregoryValente, Manuel AlmeidaK. Karabagias, IoannisValentino Kaneti, YusufKaczmarek, BeataValiente, RafaelKaczmarek, SławomirValiyaveettil, SureshKadam, AbihijitVallejo, Javier P.Kadam, AvinashValli, LudovicoKadokawa, Jun-ichiValsami-Jones, EugeniaKador, LotharVan Der Mei, Henny C.Kafarski, PawelVan Hoa, NguyenKahng, Yung HoVan Vliet, Kim M.Kaichev, V. V.Vanderbemden, PhilippeKailasa, Suresh KumarVanderzande, DirkKakati, NitulVanetsev, AlexanderKalanur, Shankara SharanappaVang-Dings, Kieng BaoKalas, WojciechVapniarsky Arzi, NataliaKalbacova, MarieVapnik, YevgenyKalish, AndreyVaradwaj, Pradeep RisikrishnaKaliva, MariaVaranasi, Venu G.Kalkan, A. KaanVarga, RastislavKallioniemi, ElisaVarlas, SpyridonKalmár, JózsefVarma, RajenderKalosakas, GeorgeVarna-Pannerec, MarianaKalozoumis, PanayotisVashurin, ArturKalska-Szostko, BeataVasile, Bogdan StefanKaltsas, GrigorisVasilev, EvgeniiKaluža, LuděkVasiliu, CristinaKalyvas, NektariosVasil’kov, Alexander YuKamada, KaiVasu, DhananjayanKamali, MohammadrezaVaszilcsin, NicolaeKamanina, NataliaVatin, NikolaiKamińska, AgnieszkaVattikuti, S. V. PrabhakarKaminski, KamilVaughey, JohnKaminski, PiotrVazquez, ManuelKamitsos, EfstratiosVázquez-Guardado, AbrahamKamnev, AlexanderVedyagin, Aleksey A.Kamp, MarlousVega Reyes, FranciscoKan, Chi-waiVeintemillas-Verdaguer, SabinoKanapitsas, AthanasiosVeis, MartinKanda, KazuhiroVejpravova, JanaKando, MasakiVejpravová, Jana KalbacovaKang, GuminVekas, LadislauKang, HyoVekinis, GeorgiosKang, Jin-Kyuvelasco ortega, eugenioKang, Kyung-TaeVelea, AlinKang, MyungkooVelikova, VioletaKang, Sang MoVelíšek, JosefKang, Sung BumVelmuzhov, AlexanderKang, Sung GuVelo Gala, InmaculadaKang, SungminVeloso, SérgioKang, TaejoonVenanzi, MarianoKang, WanliVenditti, IoleKang, YoungsooVener, Mikhail V.Kaniyoor, AdarshVenkata Ramana, MudinepalliKano, ShinyaVenkateswarlu, KattaKantartzis, Nikolaos V.Vercelli, BarbaraKanyukov, EgorVerchenko, ValeriyKao, Tsung ShengVerdejo, BegoñaKapcia, Konrad JerzyVereda Alonso, Elisa IsabelKapteijn, FreekVeres, MiklosKar, ManoranjanVergaro, VivianaKarakasidis, TheodorosVerheijen, Marcel A.Karakoti, AjayVerho, OscarKaramanis, PanaghiotisVerma, PragyaKarami, BehrouzVernardou, DimitraKaran, SumantaVerona, EnricoKarapetis, StefanosVerros, GeorgeKarashanova, DanielaVertruyen, BenedicteKarasova, Jana ZdarovaVesselli, ErikKaravasili, ChristinaVialla, FabienKarbownik, IwonaVicente, Miguel A.Karczewski, GrzegorzVicente-Manzanares, MiguelKargar, FariborzVicenzo, AntonelloKargl, RupertVida, YolandaKároly, LázárVidakis, NectariosKarpichev, YevgenVidic, JasminaKarpov, S. V.Vigneswaran, DhasarathanKarpov, Victor G.Vignoli, ValerioKarri, Rama RaoVigolo, BrigitteKartopu, GirayVijayakumar, SekarKaruppasamy, K.Vijayaraghavan, VenkateshKarwowska, EwaVikulina, AnnaKasemets, KajaVilà, AnnaKashapov, Ruslan R.Vilardi, GiorgioKashif, MuhammadVilas-Boas, VâniaKashkooli, Farshad MoradiVilhena, J. G.Kaškonienė, VilmaVillacampa Naverac, María BelénKasnatscheew, JohannesVillares, AnaKasprowicz, DobrosławaVillasante, AranzazuKasprzak, ArturVillaverde, Juan JoséKassal, PetarViña, JaimeKassner, MichaelVinattieri, AnnaKaszkur, ZbigniewVincent, LudovicKatayama, KenjiVincent-Bonnieu, SebastienKatin, KonstantinViñes, FrancescKato, DaiVinnik, DenisKatunin, AndrzejVioleta, PurcarKatz, EvgenyVirgili, TersillaKatz, Jonathan I.Virt, Ihor S.Katzer, JacekVisconti, PaoloKautek, WolfgangVishnyakov, VladimirKawaguchi, YukiViskadourakis, ZachariasKawai, TadashiViter, RomanKawakita, HidetakaVitiello, MariateresaKawasaki, HideyaVitkova, VictoriaKawashima, HidekazuVitorino, CarlaKazanskiy, NikolayVitorino, RuiKazaryan, Eduard M.Vitrik, OlegKazunobu, KojimaVittadini, AndreaKazuo, UmemuraVittori Antisari, MarcoKe, Liao LiangVizureanu, PetricǎKelarakis, AntoniosVlachos, NicholasKeller, AdrianVlahopoulos, SpirosKeller, ArturoVlase, SorinKelley, DavidVlassov, SergeiKelly, HelenaVochita, GabrielaKenanakis, GeorgeVochozka, MarekKennedy, David C.Voicu, GeorgetaKenney, MitchellVoïtchovsky, KislonKenry, K.Vokoun, DavidKepinska, MartaVoliani, ValerioKeppert, MartinVolk, JanosKerman, KaganVolk, MartinKern, WolfgangVolker, KörstgensKesari, Kavindra KumarVolkova, Elena G.Kestens, V.Vollbrecht, JoachimKetov, SergeyVolodin, Alexander M.Khabashesku, Valery N.Volodin, VladimirKHADKA, Dhruba B.Volodymyr, TkachenkoKhalakhan, IvanVolosova, Marina A.Khalid, AtaVoncina, BojanaKhan, Farhan R.Vorob’ev, AlexeyKhan, Muhammad FarooqVorotnikov, Yuri A.Khan, ZiauddingVoshkin, AndreyKhandelwal, GauravVostrikova, Kira E.Khansur, Neamul HayetVoué, MichelKhattab, TawfikVouvoudi, EvangeliaKhlebtsov, Boris N.Vranes, MilanKhonina, SvetlanaVul, AlexanderKhurshid, ZohaibW. Yang, GeKhyzhun, O.Yu.Wadati, HirokiKiani, KeivanWagner, Jakob BirkedalKiazadeh, AsalWagner-Wysiecka, EwaKichenassamy, SatyanadWahab, Mohammad AbdulKidalov, SergeyWajima, TakaakiKido, Hueliton WilianWakao, MasahiroKiefer, RudolfWaki, KeikoKijenska, EwaWalach, WojciechKim, Ae RhanWallace, Gregory Q.Kim, Byoung SuhkWallentin, JesperKim, Byung SooWallis, JohnKim, Chang-HyunWalter, ThomasKim, ChuntaeWalther, ThomasKim, Dae WooWaltl, MichaelKim, DaewonWandelt, KlausKim, Dai-SikWang, BaoyiKim, DokyoungWang, Be-JenKim, Dong YeongWang, BinKim, DonghyunWang, BoxiangKim, DonginWang, ChanghongKim, DongwookWang, ChenguoKim, Gon SupWang, Chih-FengKim, HaekyoungWang, DepengKim, Han SuWang, DongKim, Hee-JoonWang, Dong HwanKim, Hee-SeokWang, FaxingKim, HongJooWang, FeiKim, Hwa-JungWang, FukeKim, HyeminWang, GongmingKim, Hyun ChanWang, HanKim, Hyun TakWang, HongchaoKim, Hyun WooWang, HuabingKim, Hyun-oukWang, HualinKim, Hyun-SikWang, JiangweiKim, Ik-HwanWang, JianmingKim, IL TaeWang, JinqingKim, JaehwanWang, JinshengKim, Jae-HwanWang, KaifaKim, JaehyunWang, KanKim, JeesuWang, KunleiKim, JehyungWang, LihuaKim, JeonghunWang, LiqiangKim, Ji-HeeWang, LuKim, JincheolWang, LudaKim, JinsooWang, MingliKim, JiwanWang, MingsongKim, Jong HoWang, Ming-XiKim, Jong HyunWang, MoranKim, JonghoWang, Myeong-HyeonKim, Joo HyunWang, Paul C.Kim, JoohanWang, PengKim, Joo-HyungWang, QiuliangKim, Jung KyuWang, QunKim, Jun-HyunWang, RongshengKim, Ki KangWang, RuiKim, Ki SuWang, Shan-LiKim, Ki-TaeWang, ShaokaiKim, Kwang‐HyonWang, Shau-ChunKim, KyeounghakWang, ShengKim, Kyo-SeonWang, Shou-GuoKim, Kyoung-HoWang, ShuaiKim, Kyoung-KookWang, SimonKim, KyoungtaeWang, SongKim, Kyung EunWang, WeiKim, Man-HoeWang, WeijiaKim, Moon IlWang, WenKim, MoonyongWang, WenjieKim, MunhoWang, XiangxianKim, Myung-KiWang, XiaobingKim, MyungwoongWang, XiaofengKim, Sang WooWang, XinKim, Sang-JaeWang, XinhongKim, SeokWang, XinZhenKim, SeokminWang, XiupengKim, SooWang, XizuKim, SoojinWang, Xue-SongKim, SooyeonWang, XuewenKim, SoyounWang, YajunKim, Sung SooWang, YangKim, SungjunWang, Yan-HsiungKim, Tae-hwanWang, YanxiangKim, Woo YoungWang, YaomingKim, Woo-JaeWang, Yeong-HerKim, WookWang, Yi Kim, YeonginWang, YikaiKim, YongWang, Ying-JanKim, YonghunWang, YongqiangKim, Yong-SungWang, YuqiaoKim, Young-KukWang, Yu-QingKim, YoungminWang, ZhengbaoKimura, MutsumiWang, ZhenxinKioseoglou, GeorgeWang, ZhifengKiparissides, CostasWang, ZhiqianKiraly, LorantWang, ZhuangKireev, DmitryWang, ZongbaoKirillin, MikhailWarwick, Michael E. A.Kirillov, AlexanderWasilewski, TomaszKirk, AndrewWasisto, Hutomo SuryoKirstein, StefanWatanabe, KenKiryukhantsev-Korneev, Ph.V.Watanabe, MotonoriKisielewska, AnetaWatarai, HitoshiKiskinova, MayaWaterhouse, AnnaKiss, ImreWatzky, MurielleKiss, JánosWawrzkiewicz, MonikaKistanov, AndreyWdowin, MagdalenaKisu, KazuakiWeber, BirgitKita, HidekiWeber, MatthieuKitahama, YasutakaWei, DerhsinKitazawa, TakafumiWei, GangKitazumi, YukiWei, Hsien-HungKittakoop, PrasatWei, HuaKittel, AgnesWei, JunchaoKityk, Andriy V.Wei, LiKivshar, Yuri S.Wei, QiangKlang, VictoriaWei, QiupingKlapetek, PetrWei, Yu JieKlar, AgnesWeinstein, JuliaKleemann, WolfgangWeiss, RichardKleier, Daniel A.Wen, ChuangKlimm, DetlefWen, JianmingKlini, ArgyroWen, LeiKliros, George SWen, ShihuiKlos, Jaroslaw W.Wen, YangpingKnyazev, Yu.V.Wen, YuhuaKo, Fu-HsiangWenger, ChristianKo, Hyewon Wesselinowa, Julia M.Ko, SeunghwanWhitehead, Debra E.Ko, Tsung-ShineWia̧cek, Agnieszka EwaKobayashi, RikaWIbowo, Rachmat AdhiKoblischka, Michael R.Wie, Jeong JaeKobtsev, SergeyWieczorek, Władysław G.Kocbek, PetraWiegand, ThomasKocik, MarekWiekhorst, FrankKoen, BoikoWiemann, MartinKoepke, CzesławWiglusz, Rafał J.Koga, YoshikatsuWilde, JürgenKogan, EugeneWilding, MartinKohl, Yvonne LydiaWilkening, MartinKöhler, KarstenWilson, Andrew J.KOINKAR, PANKAJWilson, Justin J.Koizumi, HiroyasuWilts, BodoKojima, ChieWiltshire, Benjamin DanielKojima, ToshikatsuWindholz, LaurentiusKolaric, BrankoWiśniewski, MarekKolasinski, Kurt W.Wiśniowski, PiotrKolek, AndrzejWittemann, AlexanderKolesnichenko, Vladimir L.Włodarczyk, RenataKolev, SvetoslavWlokas, IrenaeusKolmychek, Irina A.Wojciechowski, KamilKołodyńska, DorotaWojciechowski, SzymonKołtunowicz, Tomasz NorbertWojcieszak, RobertKolychev, Eugene LWojnar, PiotrKomarov, Pavel V.Wojnarowicz, JacekKomeda, TadahiroWojnicki, MarekKomelj, MatejWojtkowska, MałgorzataKomogortsev, SergeiWolarz, ErykKomsa, Hannu-PekkaWolny, Juliusz A.Koneracká, MartinaWołowicz, AnnaKong, BiaoWong, BryanKong, KyungilWong, Danny K. Y.Kong, LingbingWong, Lai MunKong, XiangguiWong, Matthew S.Konishi, KuniakiWong, WingKononenko, Oleg V.Woon, Wei-YenKonopka, IwonaWoźniak, JarosławKonstantaki, MariaWright, C. DavidKonstantinov, Spiro M.Wu, ChaolingKontogiorgis, ChristosWu, ChunshengKontonasaki, EleanaWu, E.Kool, Leo J SchultzeWu, FeixiangKoos, Antal A.Wu, Fu-GenKopeček, JaromírWu, GuangleiKopel, PavelWu, GuoqiuKoptyug, AndreyWu, Gwo-MeiKorchowiec, BeataWu, Han-ChunKordás, KrisztianWu, HongjingKorili, Sofia A.Wu, Hsyueh-LiangKorneev, DenisWu, Jing-YunKorolkov, IlyaWu, JinmingKorona, KrzysztofWu, Jyh-MingKőrösi, LászlóWu, LeiKorri-Youssoufi, HafsaWu, LihuaKorshunov, Maxim M.Wu, MengKoruza, JurijWu, Ming-ChungKorzhikov-Vlakh, ViktorWu, MingjieKorznikova, ElenaWu, Pei-HsunKosa, GaborWu, Peter K.Kosch, OlafWu, Pin-ChiehKosionis, SpyridonWu, Shin-TsonKoslowski, ThorstenWu, WeiKosmulski, MarekWu, XiangweiKosterov, AndreiWu, XiaofengKostopoulou, AthanasiaWu, YanKotakoski, JaniWu, YingyingKotek, JanWu, YupingKotomin, Eugene A.Wu, Zhen-YuKotsilkova, RumianaWu, ZhongKotsuchibashi, YoheiWu, Zhong-ShuaiKotz, FrederikWuethrich, AlainKoubiti, MohammedWunderlich, WilfriedKoutselas, IoannisWurster, StefanKoutsokeras, LoukasWybraniec, SlawomirKoutzarova, TatyanaWyman, IanKouzes, RichardWysokinski, Karol IzydorKováč, JozefWysokowski, MarcinKovacheva, DanielaWyszkowski, MirosławKovacs, GaborXia, ChangleiKovács, ZoltánXia, DahaiKovács, ZsoltXia, GuanglinKovalcikova, AlexandraXia, MinggangKovalenko, SergeyXia, XinhuiKovalevsky, Andrei V.Xianyu, YunleiKowalczyk, AgataXiao, BingKowalczyk, TomaszXiao, BoKowalik, PawełXiao, BoqiKowalska, EwaXiao, ChunshengKowerdziej, RafałXiao, HuiningKozak, MartinXiao, JieKozakiewicz, AnnaXiao, ShuminKozhevnikov, IvanXiao, ShuyuanKozlova, Ekaterina A.Xiao, XuKozlovskiy, ArtemXie, ChaomingKozma, GáborXie, ChenKozyukhin, Sergey A.Xie, GuoxinKrajewski, MarcinXie, HongfengKrakhalev, Mikhail N.Xie, KefengKralj, SamoXie, Kelvin Y.Kralj-Iglic, VeronikaXie, QinxingKralj-Iglič, VeronikaXing, ZhicaiKrálovec, KarelXiong, HaoKramberger, ChristianXiong, Hong-bingKramer, DanielXiong, RanhuaKrasheninnikov, ArkadyXiong, RuiKrasnov, Vladimir MXu, Ben B.Krasovskii, EugeneXu, BinKratochvilova, IrenaXu, ChengKratzer, MarkusXu, ChunKraus, LuděkXu, Gui-ShengKrause, Hans-JoachimXu, HengyiKrawczynska, Agnieszka TeresaXu, HuijinKrawiec, MariuszXu, JianKrebsz, MelindaXu, JianlongKrichevskaya, MarinaXu, JiawenKristl, MatjažXu, JingjunKrisyuk, VladislavXu, Jin-ShiKritikos, GeorgiosXu, JinzeKroker, StefanieXu, LeiKrólicka, AgnieszkaXu, MinyiKrólicka, AleksandraXu, QinzhiKroll, PeterXu, QuanKronek, JurajXu, RongKruczala, KrzysztofXu, RuiKrug, Susanne M.Xu, TaoKrüger, JörgXu, Wen TaoKruit, PieterXu, WenwuKrumeich, FrankXu, XianchenKrusin-Elbaum, LiaXu, XijunKrylov, Alexander S.Xu, Xingtao Krysinski, PawelXu, XinhuaKrztoń-Maziopa, AnnaXuan, ShouhuKrzywiecki, MaciejXuan, TongtongKrzyzowska, MalgorzataXue, PengchongKuang, QinXue, XingjianKubala, LukášXue, ZhaohuiKubát, PavelXue, ZhonghuaKubo, MasatakaYablonsky, Gregory S.Kubo, YuimaruYacaman, Miguel JoseKucharska, EdytaYadav, VivekKuchmizhak, AleksandrYagi, HiroshiKuchta, BogdanYaish, Yuval E.Küçüköz, BetülYakhvarov, DmitryKudelski, AndrzejYakimova, LuidmilaKudryashov, Sergey I.Yakimova, RositsaKuila, TapasYakunin, AlexanderKujawski, WojciechYamada, MichioKukli, KaupoYamada, ShigeyukiKuklov, AnatolyYamada, YusukeKulda, VlastimilYamaguchi, AkiraKulik, TadeuszYamaguchi, MakotoKulinich, SergeiYamazaki, TomohikoKulinowski, PiotrYan, BinKulkarni, AshishYan, DayunKumar, Annamalai SenthilYan, JiahaoKumar, ArkadeepYan, ShoukeKumar, GirishYan, XuKumar, ManishYanagida, TakeshiKumar, PawanYang, BaiKumar, PrasunYang, BinKumar, RajeshYang, ChanghongKumar, SanjeevYang, Chia-MinKumar, SuhasYang, Chii-RongKumar, Vijay BhooshanYang, DapengKumar, VineetYang, DongfangKumaravel, VigneshYang, GuangKümmell, TilmarYang, HaifengKumru, BarisYang, HaoKunal, PranawYang, HuaKunc, JanYang, Hung-WeiKung, Chung-WeiYang, JunKunoh, TatsukiYang, LexianKunsagi-Mate, SandorYang, MinKunsági-Máté, SándorYang, Min YangKunwar, SundarYang, Min-QuanKupka, TeobaldYang, MinyangKurajica, StanislavYang, Po-ChihKurakevych, Oleksandr O.Yang, RuiguoKuramoto, MasaruYang, Se-RanKurlyandskaya, GalinaYang, ShikuanKurniawan, Setyo BudiYang, ShizeKurniawan, Tonni AgustionoYang, SungwooKuroda, TakashiYang, Ta-IKurpisz, MaciejYang, WenzhiKurzydłowski, KrzysztofYang, XiaofeiKushibiki, ToshihiroYang, XiaohuKusior, AnnaYang, XiaoyeKusko, MihaelaYang, XinhuaKuśtrowski, PiotrYang, XuelinKusukawa, TakahiroYang, YingpingKusuma, Victor A.Yang, YingweiKuthati, YaswanthYang, YongKuttner, ChristianYang, ZequnKuzhir, PolinaYang, ZhenzhenKuzmin, LeonidYang, ZhiKuznetsov, Vladimir L.Yang, ZhijunKuznetsova, Iren E.Yanilmaz, MeltemKwak, MinseokYannopoulos, SpyrosKwan, Hiu-yeeYao, BaichengKwela, JurekYao, BinKwiatkowski, MirosławYao, Koffi PierreKwon, Hyuck-InYao, RihuiKwon, Hyuk-JunYaraki, Mohammad TavakkoliKwon, JaeSungYaremchenko, AlekseyKwon, Kye-SiYarzhemsky, VictorKwon, Seong JungYasin, GhulamKwon, Soon-HongYastrebov, SergeyKwon, WoosungYasui, KyuichiKwon, YongwooYe, EnyiKwon, Young-WanYe, FangfuKyhm, KwangseukYe, QiangKyritsis, ApostolosYe, YunpengKyzas, George Z.Yeh, Min-HsinLa Deda, MassimoYeh, Yin-TingLa Mendola, DiegoYehezkeli, OmerLa, Duc DuongYeom, JunghoonLaaksonen, PäiviYerushalmi-Rosen, RachelLafontaine, DenisYeum, JeongLaguna, Oscar H.Yi, YougenLahiri, AbhishekYi, Young-SuLai, Chian-HuiYi, ZaoLai, Chin WeiYilmaz, Catalina Natalia CheaburuLai, Gwo-SungYilmaz, MehmetLai, Wei-ChihYim, Chae-HoLai, Ying-ChihYim, ChangyongLai, YuekunYin, KaiLaidani, Nadhira BensaadaYin, XuesongLakhno, Victor DmitrievichYochelis, ShiraLakshminarayana, GandhamYokose, SatoshiLalbakhsh, AliYokota, ShingoLalitkumar, Parameswaran GraceYokoyama, KazushigeLam, RaymondYonezu, AkioLambin, PhilippeYoo, Dong JinLamouroux, EmmanuelYoo, HyundeogLamperti, AlessioYoo, JeeyoungLan, Wen-HowYoo, KicheonLan, YuchengYoo, Yeong-MinLandi, GianlucaYoon, HyeonseokLanger, Jerzy JYoon, Jeong-wonLangner, MarekYoon, JinhoŁapińska, AnnaYoon, Jong-GulLara, AvierYoon, YeojoonLargeteau, AlainYoshida, KentaroLarsson, KarinYoshimura, MashamichiLasagni, MarinaYoshino, KenjiLasalvia, MariaYoshioka, Ken-ichiLash, Timothy D.Yoshioka, YasuoLasseuguette, ElsaYoung, JoshuaLaszlo, IstvanYoung, Kyu JeongLászló, ZsuzsannaYousefi, RaminLathiotakis, Nektarios N.Yu, Cheng-JuLatorrata, SaverioYu, ChengzhongLatterini, LoredanaYu, Deng-GuangLaurenti, EnzoYu, FabiaoLavela, PedroYu, GuanghuiLavoine, NathalieYu, HailiangLaw, Cheryl SuwenYu, Ing-SongLazar, Adriana VulpoiYu, JingangLázár, KárolyYu, LingLazar, M.Yu, LuLazarević-Pašti, TamaraYu, NuoLazzara, GiuseppeYu, Shu-QinLazzari, MassimoYu, XingeLe Breton, Jean-marieYu, XuefengLe Lagadec, RonanYu, ZhigenLe Ouay, BenjaminYuan, Chi-TsuLe Thai, DuyYuan, Chiun-JyeLe, Tien-ThinhYuan, ShaojunLeandro, PaulaYudasaka, MasakoLeavesley, DavidYudin, VladimirLebeau, BénédicteYuldashev, Sh.Lebedev, Alexander AlexandrovichYulin, AlexeyLebel, OlivierYumura, ShigehikoLeca, VictorYun, ChanghunLeclère, PhilippeYung, Ka-FuLecomte, PhilippeYurii Aleksandrovich, EreminLedda, MarioYusenko, Kirill V.Lee, ChangguZablotskii, VitaliiLee, Chang-WonZabotnov, StanislavLee, Cheng-ChungZaccone, AlessioLee, Chia-HungZach, RyszardLee, Chih-HaoZaharescu, TraianLee, Chi-ShenZaharia, CatalinLee, DaehoZahoor Raja, Muhammad AsifLee, Dai-SooZairov, RustemLee, Do NamZak, AllaLee, Dong UnŻak, KrzysztofLee, DonghyunZakaria, Mohamed B.Lee, DoojinZakharov, Alex V.Lee, Duck HyunZaki, TamerLee, Geon JoonZalas, MaciejLee, Gwan-HyoungZalden, PeterLee, GyudoZallo, EugenioLee, Hak LaeZaltariov, Mirela-FernandaLee, Han EolZaman, MohammadLee, HangilZambon, AlfonsoLee, Hee ChulZambon, DanielLee, Hee YoungZamchiy, AlexandrLee, Hiang KweeZampetti, EmilianoLee, IlkeunZanacchi, Francesca CellaLee, Jae SungZanatta, MarcileiaLee, JaehanZandvliet, HaroldLee, JaejongZanelli, Cleslei FernandoLee, Jae-SeungZang, ZhigangLee, Jin WooZangari, GiovanniLee, JinhoZannier, ValentinaLee, Jong-KwonZannotti, MarcoLee, Joon-HwaZanotto, SimoneLee, JoonseokZaraska, LeszekLee, Jung SeungZarrabi, AliLee, JunseopZarrabi, Ferdows B.Lee, Kee HagZarrin, HadisLee, Kee-SunZarzycki, ArkadiuszLee, Kyoung G.Zatsepin, Anatoly F.Lee, Man-JongZavašnik, JanezLee, Mei-hwaZavoianu, RodicaLee, MinbaekZawadzka, AnnaLee, Ming-TsangZebrev, GennadyLee, Pee-YewZec, NebojšaLee, SanghunZekker, IvarLee, Seok WooZelenovskiy, Pavel S.Lee, SeunghyunZelkó, RománaLee, Seung-JoonZeng, BaoqingLee, SunghunZeng, FaweiLee, TuZeng, LeyongLee, Wen-YaZeng, QiangLee, WookjinZeng, WenLee, Woo-KyungZeng, XiangkangLee, Ying-ChiehZeng, XianLinLee, YoungkwanZeng, YujiaLee, Yu-HsuanZgură, IrinaLee, Yun-SangZhai, TianruiLee, ZonghoonZhan, HaifeiLeem, GyuZhan, KaiyunLeem, Jung WooZhan, TaoLefkidis, GeorgZhang, BaichengLegrand, François-XavierZhang, ChengLehmkühler, FelixZhang, ChunfuLei, MingZhang, DamingLei, XiaodongZhang, FanLei, YanhuaZhang, FujunLeinartas, KonstantinasZhang, GuigangLekakou, ConstantinaZhang, HaoLekki, JanuszZhang, HongliangLeklou, NordineZhang, HuiqiLellouche, Jean-PaulZhang, JianyongLemli, BeataZhang, JiaweiLeng, YuxinZhang, JinbaoLengalova, AnezkaZhang, JunLeo, GabriellaZhang, JunyingLeon, LorraineZhang, KaiLéonard, EstelleZhang, KunLeonat, LuciaZhang, LanyingLeone, GemmaZhang, LeiLeonidas, PalilisZhang, LiuyangLeonov, AndreyZhang, MingchaoLepadatu, Ana-MariaZhang, MingzheLeporatti, StefanoZhang, QiangLepore, MariaZhang, QiangzheLeprince-Wang, YaminZhang, QianqianLesiuk, GrzegorzZhang, QichunLesnyak, VladimirZhang, QingzheLesyk, Roman B.Zhang, RuiyongLeszczyńska, AgnieszkaZhang, ShouhaiLétoublon, AntoineZhang, SonglinLettieri, StefanoZhang, TaoLevchenko, IgorZhang, TengLevina, AvivaZhang, TieruiLevine, Mindy S.Zhang, WeiLew Yan Voon, Lok C.Zhang, XiangLewandowska, KatarzynaZhang, XianghuiLewinski, JanuszZhang, XiaonanLezama, LuisZhang, XiaoxiaoLeżańska, MariaZhang, XiaoyanLi Voti, RobertoZhang, XiaoyongLi, Chia-LinZhang, XuanLi, DawenZhang, XuefengLi, DelongZhang, XuejinLi, FangsenZhang, YiLi, FengZhang, YunLi, FenghuaZhang, ZaoliLi, FujunZhang, ZhenglongLi, GuangliZhang, ZhenyiLi, GuangyuanZhang, ZhidongLi, GuohuiZhao, DeweiLi, GuoshengZhao, DongdongLi, HeZhao, DonglinLi, HongguangZhao, DongmeiLi, HongxiZhao, GuanghuaLi, HuafangZhao, GuixiaLi, IsaacZhao, JinLi, JiantongZhao, JinpingLi, JiashenZhao, LeiLi, JinganZhao, LianfengLi, JingkunZhao, MinghaoLi, JinhuaZhao, ShanLi, JinliangZhao, SikaiLi, JiushengZhao, SongruiLi, JunhuiZhao, Tian-ShengLi, JunjieZhao, WeigangLi, JunshuaiZhao, YapuLi, LanZhao, YonghaoLi, LezhongZhao, YongxiangLi, LiZhao, YudaLi, MeichengZharkov, SergeyLi, MeichunZhdanov, Andrey P.Li, MingZheng, BoLi, MingxingZheng, GongLi, MingzhongZheng, PengLi, PingZheng, XintingLi, ShiboZhidkov, Ivan S.Li, ShunboZhigang, ChenLi, ShuwenZhou, HaiboLi, SumingZhou, John L.Li, TianlongZhou, KunLi, WeiguoZhou, LiLi, WeiminZhou, PengLi, XiangnanZhou, QiangLi, Xing’aoZhou, ShengjunLi, XinleiZhou, TianhuaLi, XiuyanZhou, WeiLi, YangZhou, XiaohongLi, YiwenZhou, YanboLI, YonggangZhou, YanguangLi, YuhangZhou, YiqunLi, YujingZhou, YongLi, YunlongZhou, YouLi, ZhenhuaZhou, YuekuanLi, ZhenjiangZhou, YufengLi, ZhimingZhou, ZhuxianLi, ZongtaoZhou, ZuowanLi, ZongzeZhu, BangshangLiagre, BertrandZhu, ChenLiang, LihongZhu, DazhangLiang, QijieZhu, HanxingLiang, XingguoZhu, HaoLiang, Yuan-ChangZhu, HongyangLiao, JiaxuanZhu, HuaLiao, MeiyongZhu, JiantingLiao, XiaozhouZhu, JiqinLiao, XinqinZhu, KaiLiao, Ying-ChihZhu, LaipanLieder, MarekZhu, LipingLikodimos, VlassiosZhu, MaiyongLikozar, BlažZhu, MeihuaLillo-Ródenas, Maria ÁngelesZhu, MingqiangLim, Dong ChanZhu, QinggongLim, Dong HaZhu, ZhiweiLim, Ki-TaekZhuang, JianleLim, Lee WeiZhuang, XumingLin, BinZhuo, LongchaoLin, Cheng-TeZhuravlev, VictorLin, Chien-hongZhuravlev, Yurii N.Lin, Chun-RongZianni, XanthippiLin, CunguoZicans, JanisLin, Dan JaeZidanšek, AleksanderLin, DuoZidovec Lepej, SnjezanaLin, HongzhenZielasek, VolkmarLin, Hung-YinZielinska, AleksandraLin, MingZielinska, BeataLin, Pao TaiZielinski, AndrzejLin, Shuo ParkZielinski, PiotrLin, Wei-NingZille, AndreaLin, Yang-weiZilm, PeterLin, YuankunZimbone, MassimoLinares, N.Zimmermann, UweLindström, TomZimnyakov, DmitryLing, YunZinicovscaia, IngaLinkous, Clovis A.Zinna, FrancescoLionetto, FrancescaZiora, ZytaLiopo, AntonZiyatdinova, GuzelLiotta, Leonarda FrancescaZobi, FabioLipparini, FilippoZoccatelli, GianniLipphaus, AndreasŽoldák, GabrielLipsanen, HarriZolnai, ZsoltLis Arias, Manuel J.Zoltán, PásztiLis, Jose Vicente RosZota, Cezar B.Lisa, GabrielaZou, HaiyangLisco, FabianaZou, HongyanLisi, LucianaZou, YoushengLisi, NicolaZoubi, Wail AlLisinska-Czekaj, AgataZozulya, AlexeyLisjak, DarjaZubavichus, Yan V.Lisowska-Oleksiak, AnnaZubrik, AntonListorti, AndreaZucchelli, AndreaLitvak, AlexanderZukalová, MarkétaLitvinchuk, AlexanderZukowski, WitoldLiu, BaiquanZuo, ChuantianLiu, Bo-TauZuo, JianLiu, Chao-LinZuorro, AntonioLiu, Chee-WeeZverev, Vladimir I.Liu, Chung-ChiunZvereva, Irina A.Liu, Chung-JungZytkiewicz, Zbigniew


